# Single Indium Atoms and Few-Atom Indium Clusters Anchored onto Graphene via Silicon Heteroatoms

**DOI:** 10.1021/acsnano.1c03535

**Published:** 2021-08-19

**Authors:** Kenan Elibol, Clemens Mangler, David D. O’Regan, Kimmo Mustonen, Dominik Eder, Jannik C. Meyer, Jani Kotakoski, Richard G. Hobbs, Toma Susi, Bernhard C. Bayer

**Affiliations:** † University of Vienna, Faculty of Physics, Boltzmanngasse 5, A-1090, Vienna, Austria; ‡ Centre for Research on Adaptive Nanostructures and Nanodevices (CRANN) and the SFI Advanced Materials and Bio-Engineering Research Centre (AMBER), Dublin 2, Ireland; § School of Chemistry, Trinity College Dublin, The University of Dublin, Dublin 2, Ireland; ∥ School of Physics, Trinity College Dublin, The University of Dublin, Dublin 2, Ireland; ⊥ Institute of Materials Chemistry, Vienna University of Technology (TU Wien), Getreidemarkt 9/165, A-1060 Vienna, Austria; # Institute for Applied Physics, University of Tübingen, Auf der Morgenstelle 10, 72076 Tübingen, Germany

**Keywords:** single atoms, nanoclusters, 2D materials, graphene, anchoring, aberration-corrected scanning transmission electron microscopy

## Abstract

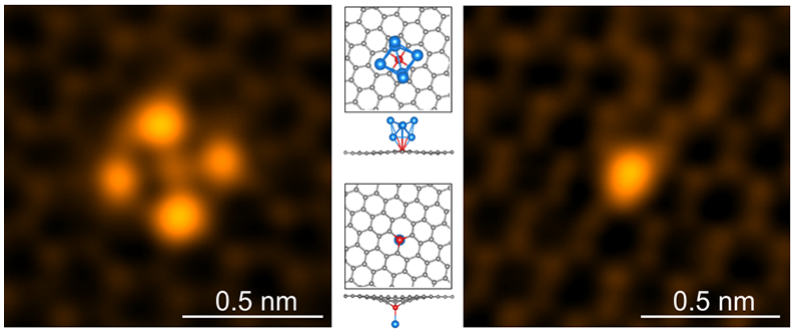

Single atoms and few-atom nanoclusters are of high interest in catalysis and plasmonics, but pathways for their fabrication and placement remain scarce. We report here the self-assembly of room-temperature-stable single indium (In) atoms and few-atom In clusters (2–6 atoms) that are anchored to substitutional silicon (Si) impurity atoms in suspended monolayer graphene membranes. Using atomically resolved scanning transmission electron microscopy (STEM), we find that the symmetry of the In structures is critically determined by the three- or fourfold coordination of the Si “anchors”. All structures are produced without electron-beam induced materials modification. In turn, when activated by electron beam irradiation in the STEM, we observe *in situ* the formation, restructuring, and translation of the Si-anchored In structures. Our results on In–Si-graphene provide a materials system for controlled self-assembly and heteroatomic anchoring of single atoms and few-atom nanoclusters on graphene.

## Introduction

Supported single atoms and atomic clusters comprising only a few atoms (“nanoclusters”) have distinct electrical, optical, magnetic, and catalytic properties. These have suggested single atoms and few-atom clusters to be of high application potential, particularly in heterogeneous catalysis (“single-atom catalysts” to “few-atom catalysts”)^1−4^ as well as in nanoplasmonics.^5^ For catalysis applications, single atoms and few-atom clusters anchored to carbon substrates are of particularly high relevance.^3,6−11^ Shortcomings in fabrication and synthesis, controlled placement and anchoring, and characterization in terms of, e.g., structure, composition, and stability, currently hinder their further study and use.

An emerging pathway toward both controllable fabrication and concurrent microscopic imaging of small atomic arrangements is the application of aberration-corrected scanning transmission electron microscopy (STEM) to atoms that are placed onto or implanted into suspended two-dimensional (2D) membranes such as graphene.^12−16^ Controlled placement of individual impurity atoms within the graphene lattice via the controlled movement of the STEM electron beam (e-beam) has been demonstrated,^17−19^ mostly relying on ubiquitous substitutional Si impurities inherent to chemical vapor deposited (CVD) graphene,^20^ which however also can be deterministically introduced.^21^ This STEM-based manipulation has been expanded toward the study of homoatomic Si clusters in graphene.^22−26^ Recently, the e-beam driven merging of such Si clusters with a single Pt atom toward heteroatomic Pt–Si structures on graphene was also reported.^27^ A shortcoming of the STEM manipulation approach is, however, the difficulty in extending it to larger-scale fabrication. A materials system that intrinsically and readily allows single-atom and few-atom cluster formation and anchoring on graphene membranes in a self-assembled fashion has remained elusive to date.

We report here the self-assembly of room-temperature-stable single In atoms (1 atom) and few-atom In clusters (2–6 atoms) and their concurrent anchoring onto individual substitutional Si dopant atoms in graphene membranes. We employ atomically resolved and element-sensitive STEM^28^ to observe the In–Si structures including probing their structural dynamics and formation mechanisms *in situ via* e-beam induced structural rearrangements.^29,30^ We find that the original coordination of Si in the graphene lattice is critical for the atomic arrangement of the In single atoms and clusters. While single In atoms and threefold symmetric In clusters form on threefold coordinated Si atoms, fourfold symmetric clusters are found on fourfold coordinated Si atoms. Within the fourfold and threefold symmetric clusters, In atoms can be located on top of either carbon atoms or hexagon centers. Hexagon-centered fourfold symmetric In_6_ clusters can dynamically transform into three different structures during e-beam irradiation: a chain with six In atoms, an In dimer located on a pentagon center, and a dimer located on a hexagon center. Unlike the fourfold symmetric clusters which are immobile under electron irradiation,^20^ the C-centered threefold symmetric In_5_ clusters can move within the graphene lattice due to the electron irradiation induced movement of the underlying threefold coordinated Si site. These threefold clusters can also transform to anchored single In atoms.

Typically foreign single atoms and nanoclusters on graphene have been found to preferentially reside at high-reactivity sites in the graphene such as lattice defects^4,31^ or adventitious carbon contamination.^32^ Self-assembled anchoring on graphene via single foreign impurity atoms in the graphene^33,34^ (such as on Si impurities here) has been theoretically suggested^21,35^ but not experimentally verified, in particular, not on the atomic scale. Toward filling this gap, our experimental observations here evidence a large diversity of room-temperature-stable, heteroatomically anchored single atoms and nanoclusters in the In–Si-graphene system. Our work thereby contributes a materials system toward the development of a rational toolbox for the controlled self-assembly and heteroatomic anchoring of single atoms and few-atom clusters on graphene.

## Results and Discussion

**Figure 1:**
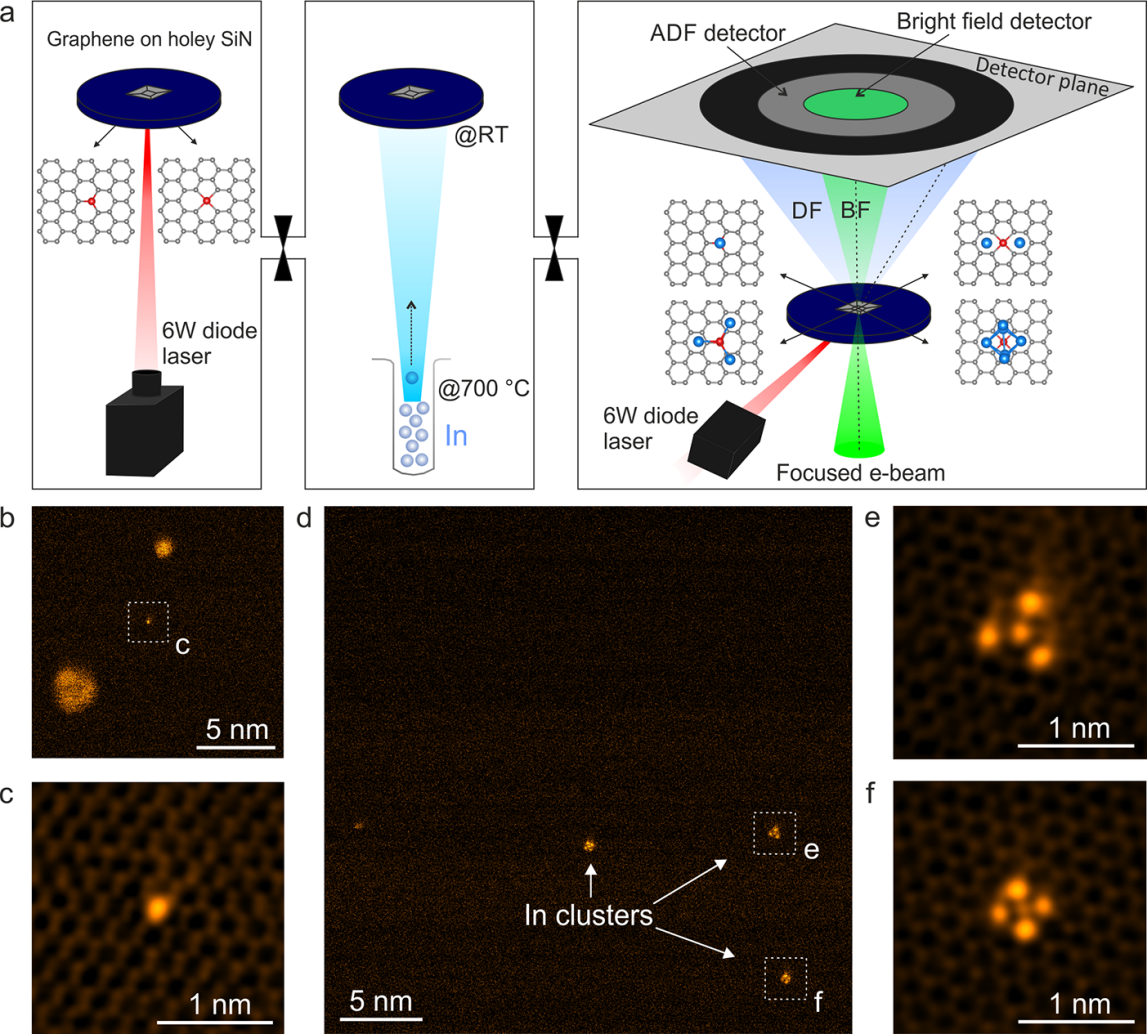
(a) Schematic illustration of the experiment involving laser irradiation, *in situ* In deposition, and imaging by STEM. The blue, red, and gray colored atoms are In, Si, and C, respectively. (b,d) Large-area MAADF-STEM images of a clean monolayer graphene surface after *in situ* deposition, showing a single In atom (b) and few-atom In clusters (d). (c,e,f) Close-up MAADF-STEM images of a single In atom and threefold and fourfold symmetric In clusters indicated by white dashed frames in (b,d). All images display false color, and images in (c,e,f) are double-Gaussian-filtered after Wiener filtering to reduce noise and enhance contrast (see Methods section; raw images are shown in SI Figure S1). Note that the contrast of the underlying graphene lattice is low compared to the clusters, making it difficult to display both clearly in some of our figures.

The experiments we carried out to deposit indium single atoms and few-atom clusters on graphene are schematically shown in Figure 1a. A monolayer CVD graphene membrane suspended over a holey SiN chip is first loaded into the STEM setup (ultrahigh vacuum (UHV) with base pressure ∼10^–9^ mbar), which comprises the microscope and directly coupled preparation chambers including laser annealing and an *in situ* evaporation chamber. We note that CVD graphene intrinsically includes a small number of substitutional Si heteroatoms, as well-documented in prior literature.^20,36,37^ The sample is then first irradiated in UHV by 600 mW of laser power at 445 nm wavelength, allowing the removal of adventitious hydrocarbon adsorbates from the graphene that stem from membrane fabrication and ambient air exposure.^38,39^ Subsequently, without vacuum break, In is evaporated onto the graphene membrane using a custom-built preparation chamber (base pressure ∼10^–9^ mbar).^30^ After *in situ* deposition of In, the sample is transferred into the microscope, without a vacuum break, and once more irradiated *in situ* by a similar 600 mW laser before STEM imaging. Due to the low melting point (∼160 °C) but low vapor pressure of In,^40^ the second laser irradiation critically promotes the diffusion of In atoms over the graphene surface.

During STEM imaging, medium and high angle annular dark field (MAADF and HAADF) signals are acquired simultaneously. These allow the discrimination of imaged elements based on signal intensity (our HAADF intensity scales with atomic number as *Z*^∼1.6^ for isolated atoms and roughly linear with specimen thickness for a given *Z*).^28^ Further elemental identification is made via simultaneously recorded electron energy loss spectroscopy (EELS) data. Further experimental details can be found in the Methods section.

Figure 1b–f shows that large atomically clean areas (between large In nanoparticles;^30^ see also SI Figure S1a) are found on the graphene after In deposition and the second laser irradiation. The key feature of interest we observe in these large clean graphene areas are the small bright spots in the MAADF images in Figure 1b–f. We identify these spots as single In atoms (Figure 1c) and few-atom In clusters formed on the graphene surface (Figure 1e,f), which are all anchored to the graphene via single substitutional Si impurity atoms covalently bound within the graphene lattice, as described in detail below. Notably for the clusters, we consistently observe two different symmetries: threefold and fourfold. (These symmetries refer to as-observed contrast within the image projection plane, as typical for (S)TEM measurements. Out-of-plane atom positions in the structures are not readily accessible by STEM, but will be elucidated based on our associated structural modeling and image simulations.)

We stress that the single In atoms and In clusters are already present before extended STEM imaging, i.e., they are generally not produced by the electron irradiation, but have self-assembled during In evaporation and the second laser anneal prior to imaging. In the fields of view in Figure 1b,d (with electron irradiation dose rates of 0.73 × 10^6^ e^–^ nm^–2^ s^–1^ (b), 0.18 × 10^6^ e^–^ nm^–2^ s^–1^ (d)) the In structures appear to be unperturbed by STEM imaging. We verify the structure of these In clusters on graphene at higher magnification in the next sections. Imaging at higher magnifications is associated with higher electron dose rates, however, which drives structural modifications of the In structures by energy transfer from the scanning e-beam,^29,30^ but at the same time it also allows us to *in situ* probe their structural dynamics.

**Figure 2:**
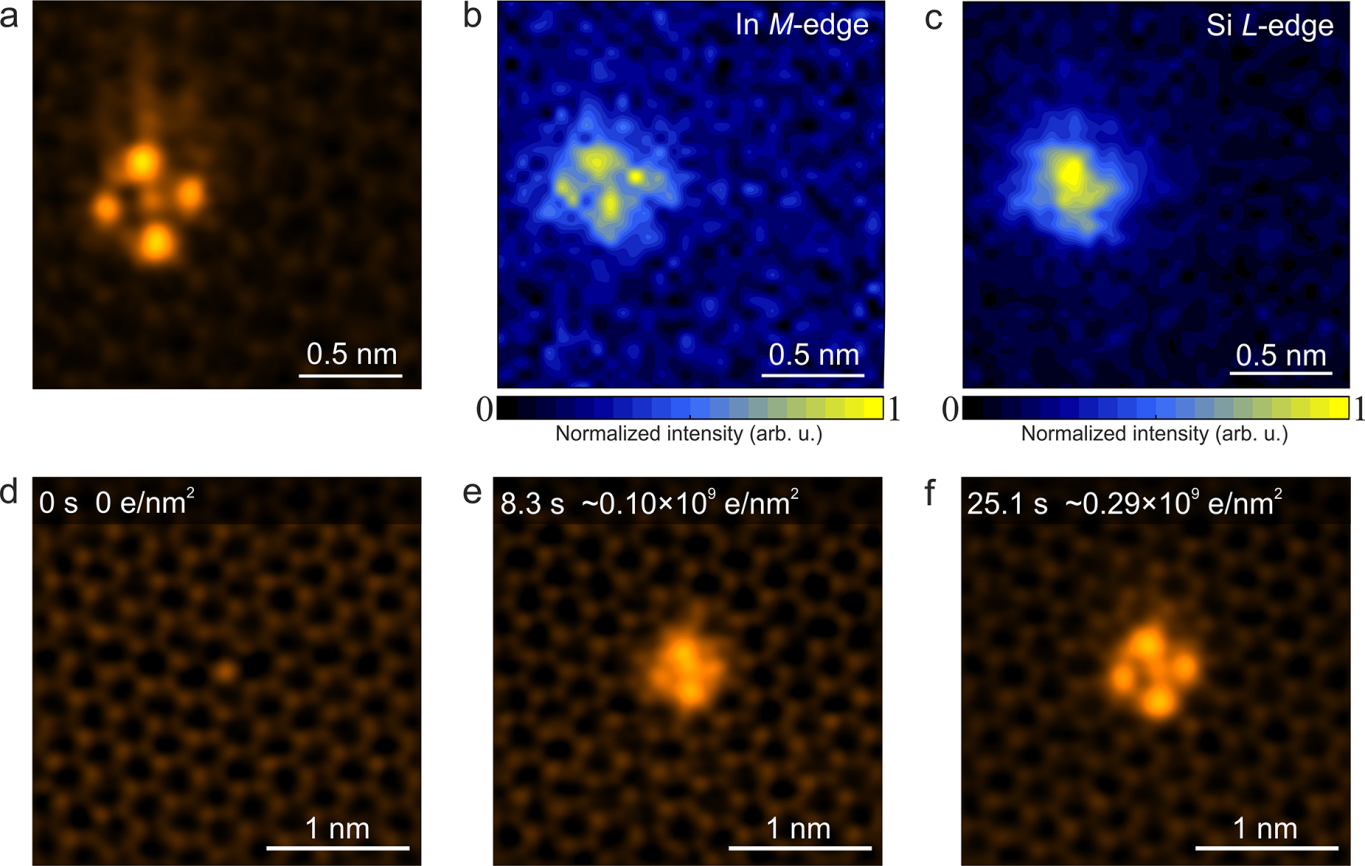
(a) HAADF-STEM image of a fourfold symmetric cluster and (b,c) its simultaneously acquired EEL spectrum maps showing the In *M*-edge (b) and the Si *L*-edge (c), respectively. (d–f) MAADF-STEM images of (d) fourfold coordinated Si impurity in graphene, (e) intermediate In structure anchored onto this Si atom, and (f) fourfold symmetric In cluster trapped on the same Si atom. All experimental HAADF and MAADF images are in false color and filtered (see Methods section; raw images are shown in SI Figure S2). For EEL spectra of the In cluster, see SI Figure S3.

To verify the chemical nature of the structures, an EEL spectrum image is acquired on a typical cluster shown in the HAADF image of Figure 2a. EELS accumulated with an energy range focused on the In *M-* and Si *L*-edges show that the cluster consists of In and Si atoms (Figure 2b,c). The EELS data indicates fourfold symmetry for the positions of In around a rather central Si signal. This is a first hint at the Si-anchoring of the In atoms on the graphene lattice. From the EELS map, however, it remains unclear if the Si signal is from one or several central Si atoms.

Toward addressing this question, the HAADF image sequence in Figure 2d–f (see also SI Video 1) reveals the self-assembly process of the In–Si structures (here emulated by the energy input from the e-beam). This sequence has been acquired at a higher magnification (and thus higher electron dose rate of 20.83 × 10^6^ e^–^ nm^–2^ s^–1^) than Figure 1b,d and thereby enables observation of e-beam induced structural dynamics.^29,30^ In particular, Figure 2d shows initially a bare fourfold coordinated Si atom in the graphene lattice. After 8.3 s (electron dose 0.1 × 10^9^ e^–^ nm^–2^ s^–1^), In atoms have become trapped at this Si atom to form an intermediate Si-anchored In structure (Figure 2e; further details on this intermediate possible dimer structure are discussed below in Figure 4). After 25.1 s (Figure 2f, electron dose 0.29 × 10^9^ e/nm^2^), additional In atoms have become trapped to eventually form a fourfold symmetric In cluster anchored to the fourfold Si atom in the graphene lattice (further details on the structure below). This observation of In cluster formation suggests that a single central Si atom is present in the observed In cluster structures. Notably, no In reservoir is present within the field of view around the Si atom during cluster formation in Figure 2d–f.^27^

Combined, Figure 1 and Figure 2 suggest a self-assembly formation mechanism for the In clusters in which In atoms or moieties diffuse across the graphene membrane (with much higher diffusion speed than resolvable by STEM^29,41^) and become trapped during encounters with the substitutional Si, thereby self-assembling into the observed Si-anchored In clusters on the graphene (with some Si atoms remaining “empty” from our processing). We suggest that this facile self-assembly relies on the significant mobility^42^ of In atoms on the graphene membrane at room temperature (resulting from In’s low melting point (∼160 °C) and low vapor pressure over a wide temperature range^40^) and the higher reactivity of the Si site compared to the relatively inert graphene lattice.^21,33−35^ The observation in Figure 2d that initially empty Si impurity sites became active toward In capture only upon the higher electron dose rate irradiation at smaller fields of view might be related to sustained ionization of the Si site that could increase its reactivity and/or act as an attractive potential for diffusing In atoms, though this possibility is difficult to quantify.^43^ For the as-produced clusters imaged at larger fields of view (as in Figure 1b–f), such an effect of electron irradiation is excluded, since the presence of all structures upon switching on the electron beam (i.e., without assembly under the e-beam) confirms that the structures observed at this field of view are not produced by electron irradiation, but rather have self-assembled during In evaporation and the second *in situ* laser anneal prior to imaging.

**Figure 3:**
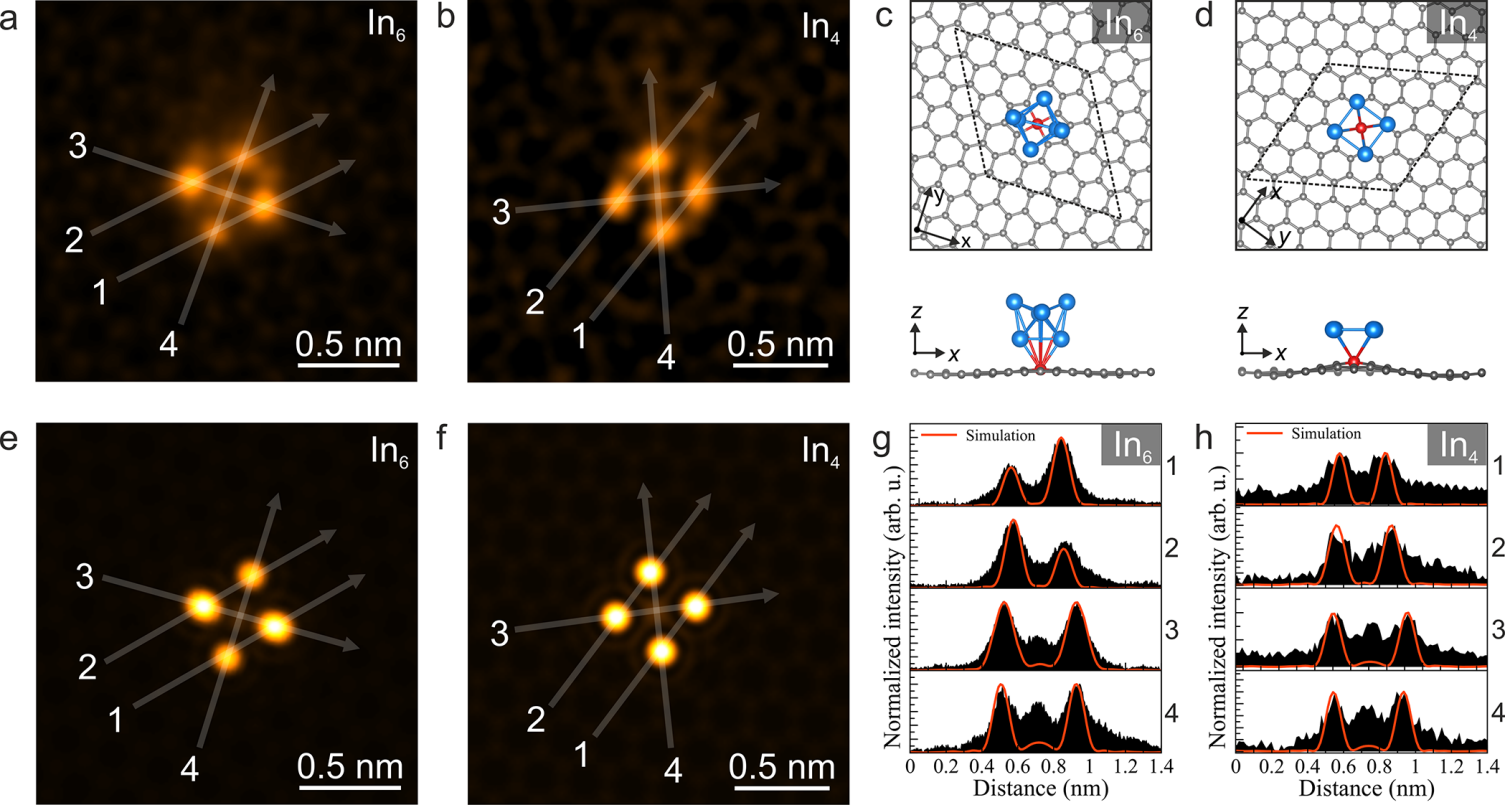
(a,b) HAADF-STEM images of hexagon-centered fourfold symmetric In_6_ and C-centered fourfold symmetric In_4_ clusters, respectively. (c,d) Plane and side views of DFT-relaxed models with six and four In atoms used in the image simulations. The dashed lines on the atomic models delineate the computational graphene 5 × 5 supercell containing the In cluster. The blue, red, and gray colored atoms are In, Si, and C, respectively. (e,f) Simulated HAADF images corresponding to the clusters shown in (a) and (b), respectively. (g,h) Intensity profiles measured along the white dashed lines on the experimental and simulated HAADF images of fourfold symmetric In_6_ and In_4_ clusters, respectively. We note that due to the unequal projected intensities for the two sets In positions in (a,c) the In_6_ cluster could in projection also be labeled as twofold instead of fourfold. All experimental HAADF images are shown in false color and are double-Gaussian-filtered (see Methods section).

After this first assessment of the overall nature of the anchored clusters, we now turn to detailed atomic structure analysis aided by density functional theory (DFT)-based structure modeling. In Figure 3, the fourfold symmetric In clusters from Figure 1f and Figure 2 are analyzed: Our data reveals that there are two different fourfold symmetric In clusters forming on fourfold coordinated Si atoms as shown in Figure 3a,b. Based on the EELS findings and the cluster formation sequence in Figure 2, we have relaxed atomistic models comprising In, Si, and C atoms with DFT to use in STEM image simulations (see Methods section). The first model in Figure 3c addresses the experimental observation that two of the In atomic columns are brighter than the other two and thus involves six In atoms anchored on a fourfold coordinated Si impurity in graphene. In this model, the In atoms are located over the hexagon centers of graphene. We thus term this type of structure a hexagon-centered fourfold In_6_ cluster. The image simulation shown in Figure 3e is in a good agreement with the experimental image (Figure 3a), as shown by the line profiles recorded over the experimental and simulated HAADF images (Figure 3g). (The intensity at the center of cluster is slightly higher in the experimental data, which we attribute to the probe tails that can cause intensity enhancement at the center surrounded by six intensely scattering In atoms.^44^ However, note that the projected atomic positions are quite sensitive to the atoms present in the cluster.)

The second observed structure is also fourfold symmetric (Figure 3b), but the experimental contrast is equal for all four In columns, indicating that there are four In atoms in the cluster all located over C atoms (see Figure 3d). We thus call this structure a C-centered fourfold In_4_ cluster. The DFT-relaxed model matches the experimental data well (see Figure 3b,f,h) (except the intensity at the center of cluster in experimental data, which is again higher compared to simulated data). Besides In atoms, we have tried atomic models including Ca, Cu, and Fe metal atoms and compared the intensities and symmetries of experimental and simulated HAADF images based on DFT-relaxed models (see SI Figure S4). Those result in a much poorer match, further corroborating that the bright spots on HAADF images are In. Besides these fourfold symmetric In clusters, we considered another In structure with four In and two Si atoms (see SI Figure S5). This structure is similar to that shown in Figure 3b, but the cluster contains one additional Si, which pushes the four In atoms away from the center and thus despite increasing the intensity of the central spot does not match the overall experimental contrast.

**Figure 4:**
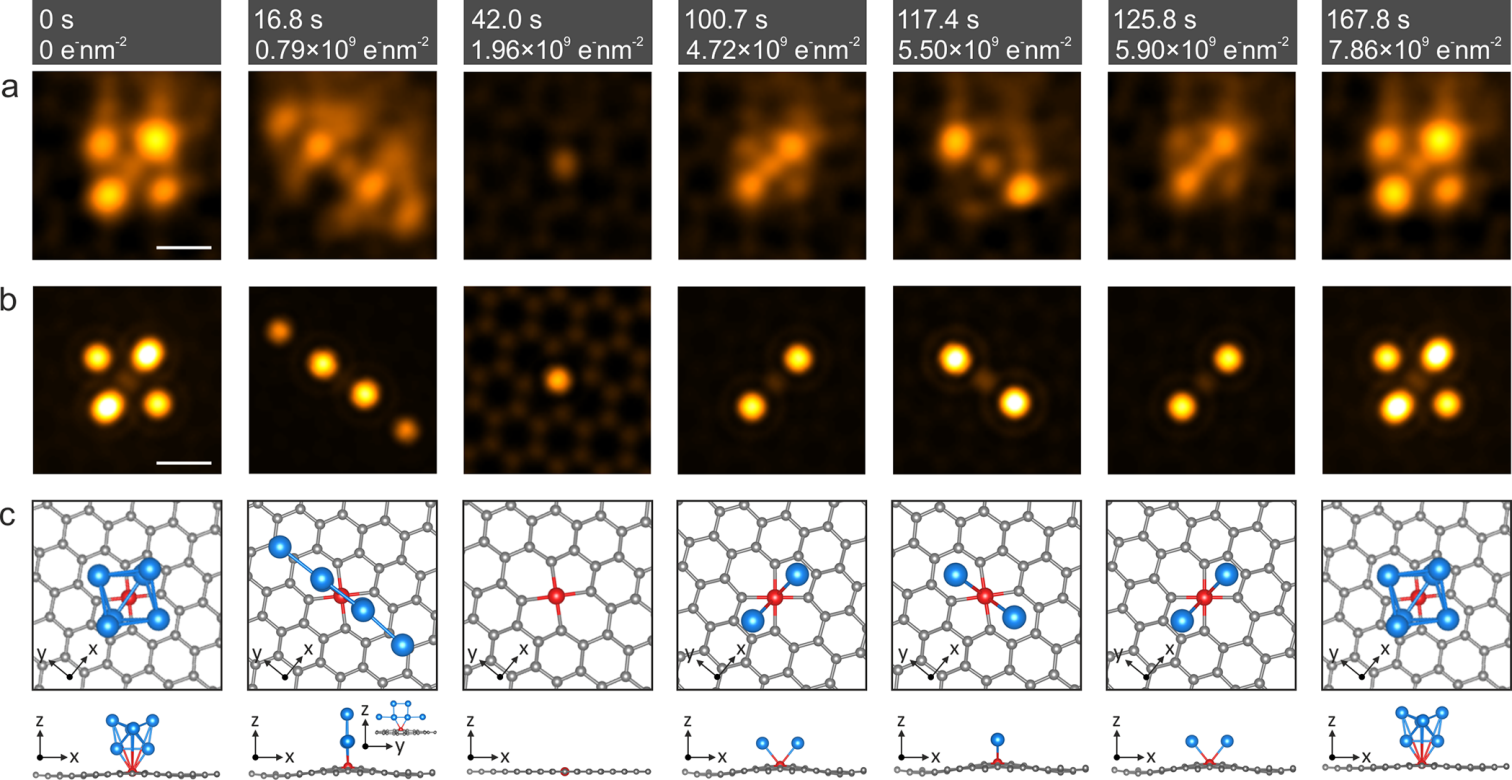
(a) HAADF-STEM images acquired at successive times, corresponding to increasing irradiation doses (indicated over the panels). (b) HAADF image simulations corresponding to the experimental images in (a). (c) Plane and side views of DFT-relaxed models with In clusters anchored on the fourfold coordinated Si impurity in graphene. All experimental HAADF images are in false color and double-Gaussian-filtered (see Methods section). The scale bars are 0.3 nm.

The dynamics of a hexagon-centered fourfold In_6_ cluster under electron irradiation (dose rate 46.88 × 10^6^ e^–^ nm^–2^ s^–1^) are shown in Figure 4a (raw images in SI Video 2). In Figure 4b,c, the simulated HAADF images and atomic models matching with the experimental HAADF images of the cluster are presented. The initial structure of this cluster with six In atoms is transformed after 16.8 s (electron dose of ∼0.79 × 10^9^ e^–^ nm^–2^) into an In chain where the In atoms are aligned along the armchair direction of the graphene lattice, with all six In atoms retained. After a further 42.0 s (∼1.96 × 10^9^ e^–^ nm^–2^), all In atoms of the cluster disappear from the field of view. Notably, a central fourfold symmetric substitutional Si impurity in the graphene lattice remains visible, again corroborating the role of substitutional Si as a central “anchor” for the In clusters on graphene. (The additional intensities around the In atoms in Figure 4 are due to the probe tail. Since Si scatters less than In, the probe tail is less visible around the Si atom in the HAADF image with relatively low contrast.) After 100.7 s (∼4.72 × 10^9^ e^–^ nm^–2^), two In atoms are again captured by the Si atom (similar as in the cluster formation sequence in Figure 2d–f) and located over the pentagon centers of the fourfold Si impurity site. After 117.4 s (∼5.50 × 10^9^ e^–^ nm^–2^); these two In atoms rotate by 90° to overlay the graphene hexagon centers. However, they return back to their initial position by again rotating 90° after 125.8 s (∼5.90 × 10^9^ e^–^ nm^–2^). This structure finally attracts four additional In atoms after 167.8 s (∼7.86 × 10^9^ e^–^ nm^–2^) to form the hexagon-centered fourfold In_6_ cluster, identical to that observed in the first frame of the sequence.

**Figure 5:**
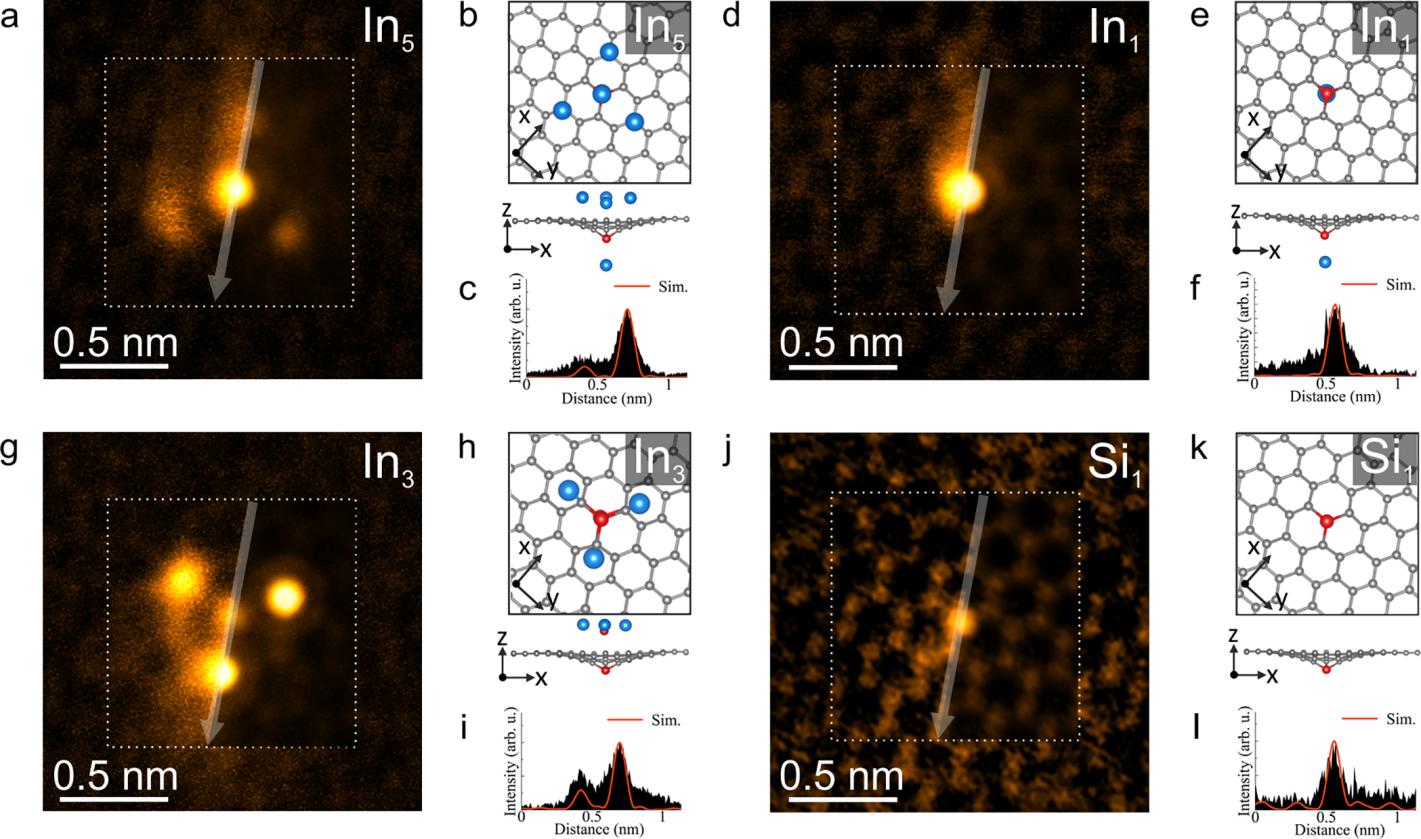
Atomic structure analysis of threefold symmetric In clusters. (a,d,g,j) HAADF-STEM images of (a) a C-centered threefold symmetric In_5_ cluster and (d) In_1_ single atom (the same area as in (a) after four In atoms are removed during electron irradiation at a dose of ∼0.29 × 10^9^ e^–^ nm^–2^) as well as (g) hexagon-centered threefold symmetric In_3_ cluster and (j) Si_1_ in graphene lattice (the same area as in (g) after three In atoms and one Si atom surrounding the anchoring Si atom are removed during electron irradiation at a dose of ∼0.39 × 10^9^ e^–^ nm^–2^), respectively. Semitransparent simulated HAADF images corresponding the structures shown in panels b, e, h, and k are shown on the right side of line profiles in white dashed frames on panels a, d, g, and j (left side of line profiles display raw image). The experimental images are in false color and Wiener filtered (raw images are shown in SI Figure S11). (b,e,h,k) Plane and side views of the DFT-relaxed models used for the image simulations shown in panels a, d, g, and j, respectively. The blue, red, and gray colored atoms shown in the atomic models are In, Si, and C, respectively. (c,f,i,l) Intensity profiles recorded along the semitransparent white lines overlaid on the experimental and simulated HAADF images in (a,d,g,j). The identity of the Si site was verified by EELS (SI Figure S7).

In addition to indium clusters formed on fourfold coordinated Si impurities, we observe threefold symmetric In clusters as well as single In atoms anchored on threefold coordinated Si impurities, as shown in detail in Figure 5. Similar to the fourfold symmetric cases, In atoms in threefold symmetric clusters may be located over the graphene C atoms or hexagon centers, and there are two distinct variants, here differing by the intensity of the central atomic column. We term these the C-centered threefold In_5_ cluster (see Figure 5a,b) and the hexagon-centered threefold In_3_ cluster (see Figure 5g,h). Notably, our structure assignment indicates that the C-centered threefold In_5_ cluster has one In atom residing on the opposite side of the graphene membrane as compared to the other In atoms (see also SI Figure S6). We suggest this to be related to In diffusion through defects or grain boundaries in the CVD graphene, that enable some In atoms to reach the graphene membrane’s opposite side under our conditions. The hexagon-centered threefold In_3_ structure notably includes two Si atoms, one of which is substitutional in the graphene lattice and one which is part of the In cluster. As shown in Figure 5a–c and g–i, the DFT-relaxed models and experimental data are in a good agreement.

Under e-beam exposure at higher magnifications, the In atoms in these threefold clusters can be readily ejected by the focused electron beam (see Figure 5a,d, and g,j, also SI Videos 3, 4, 5, and 6). HAADF-STEM images in Figure 5d show a single In atom anchored on the threefold coordinated Si impurity after the other In atoms are removed from the threefold In_5_ cluster. Notably, although four In atoms are removed, the remaining single In atom keeps its position on the Si atom under electron irradiation (see SI Videos 4, 5, and 6). In addition to the model shown in Figure 5b, we have created two different models with four and five In atoms and compared them with the experimental data (see SI Figure S6). As shown in SI Figure S6d, the atomic structure shown in Figure 5b is in a better agreement with the experimental image intensity.

Unlike C-centered threefold In_5_, in the hexagon-centered threefold In_3_ cluster only the anchoring Si impurity remains in the graphene lattice after removal of three In atoms and one Si atom (see also EEL spectra in SI Figure S7). (We attempted to relax a C-centered threefold symmetric In_3_ cluster, i.e., without an In or Si atom directly on top of the Si impurity; see SI Figure S8; but the spacing of the cluster and the intensity of the center column do not match the experimental data.)

We note that, intermittently, short-term capture of single In atoms by fourfold Si impurities is also observed (SI Figure S9), albeit these arrangements are never as stable as in the case of threefold Si anchoring. We also note that when the coordination of the Si atom in the graphene lattice is changed due to e-beam exposure, trapping of different structures on the same Si atom is observed. For instance, in SI Figure S10 a threefold symmetric In cluster is first trapped by a threefold coordinated Si atom, then again removed, after which the Si atom switches into fourfold coordination, which eventually enables the trapping of an In_2_ dimer.

After having identified the various observed structures of the In–Si-graphene system, we can semiquantitatively elucidate their relative formation probabilities by measuring their areal observation densities when imaged at wide fields of view (i.e., without inducing structural dynamics with a higher e-beam dose rate; the total number of observations was 43). Note that observed areal densities of bare fourfold coordinated Si (0.82/1000 nm^–2^) and bare threefold coordinated Si (0.89/1000 nm^–2^) anchoring heteroatoms in the graphene lattice are roughly similar for our samples. In descending order, we find the most often observed (thus, arguably, most readily formed) Si-anchored In structures to be as follows: hexagon-centered fourfold symmetric In_6_ clusters (0.37/1000 nm^–2^), single In atom anchored on Si (0.32/1000 nm^–2^), hexagon-centered In_3_ threefold symmetric clusters (0.22/1000 nm^–2^), C-centered fourfold symmetric In_4_ (0.20/1000 nm^–2^), C-centered threefold symmetric In_5_ clusters (0.15/1000 nm^–2^), In_2_ dimers (0.12/1000 nm^–2^), and In_6_ chains (0.07/1000 nm^–2^). These statistics underline that both In single atoms and few-atom clusters are well stabilized by the Si-graphene system. Notably we find neither single In atoms nor few-atom In clusters that have nucleated without Si “anchors”, such as on the empty graphene basal plane or at single vacancy or topological defects in the graphene.

The structure of the observed Si-anchored In nanoclusters is not related to the body-centered-tetragonal bulk structure of In particles, which we have previously observed for much larger In particles on graphene with average particles size of ∼5 nm.^30^ Two prior DFT works on Si-doped In clusters considered only unsupported nanoclusters.^45,46^ In these studies, terminal Si positions were found to be energetically less favorable than central Si positions. Due to the presence of the graphene in which the Si is embedded in our work, these prior studies have however only limited comparability. In the bulk, In and Si have a very limited miscibility.^47^ In SI Table 1, we provide DFT total energy calculations for the In–Si-graphene structures observed here. Interestingly, we find no clear correlation between calculated total energies per atom or formation energies of the observed structures with our experimentally observed areal observation densities. In SI Table 2, we also find that DFT predicts that adding additional In atoms to the observed structures remains energetically favorable for our cluster sizes. Thus, DFT does not elucidate why we observe only single In and few-atom In nanoclusters (2–6 atoms) anchored on the Si impurities and not larger particle formation. However, since DFT calculations address only the static stability of the structures, this points to kinetic effects in the formation of the observed structures. These effects might be related to limited In supply (which we suggest to be unlikely, given the readily observed formation of Si-anchored In clusters during our *in situ* observations) or dynamic attachment/detachment barrier differences between In–Si and In–In, respectively. The latter possibility is supported by the fact that for most of our observed structures, the majority of In atoms are on the Si’s first coordination shell (i.e., most of the In atoms are bound to the Si anchor), while purely In-bound In atoms remain limited in our experimentally observed structures.

**Figure 6:**
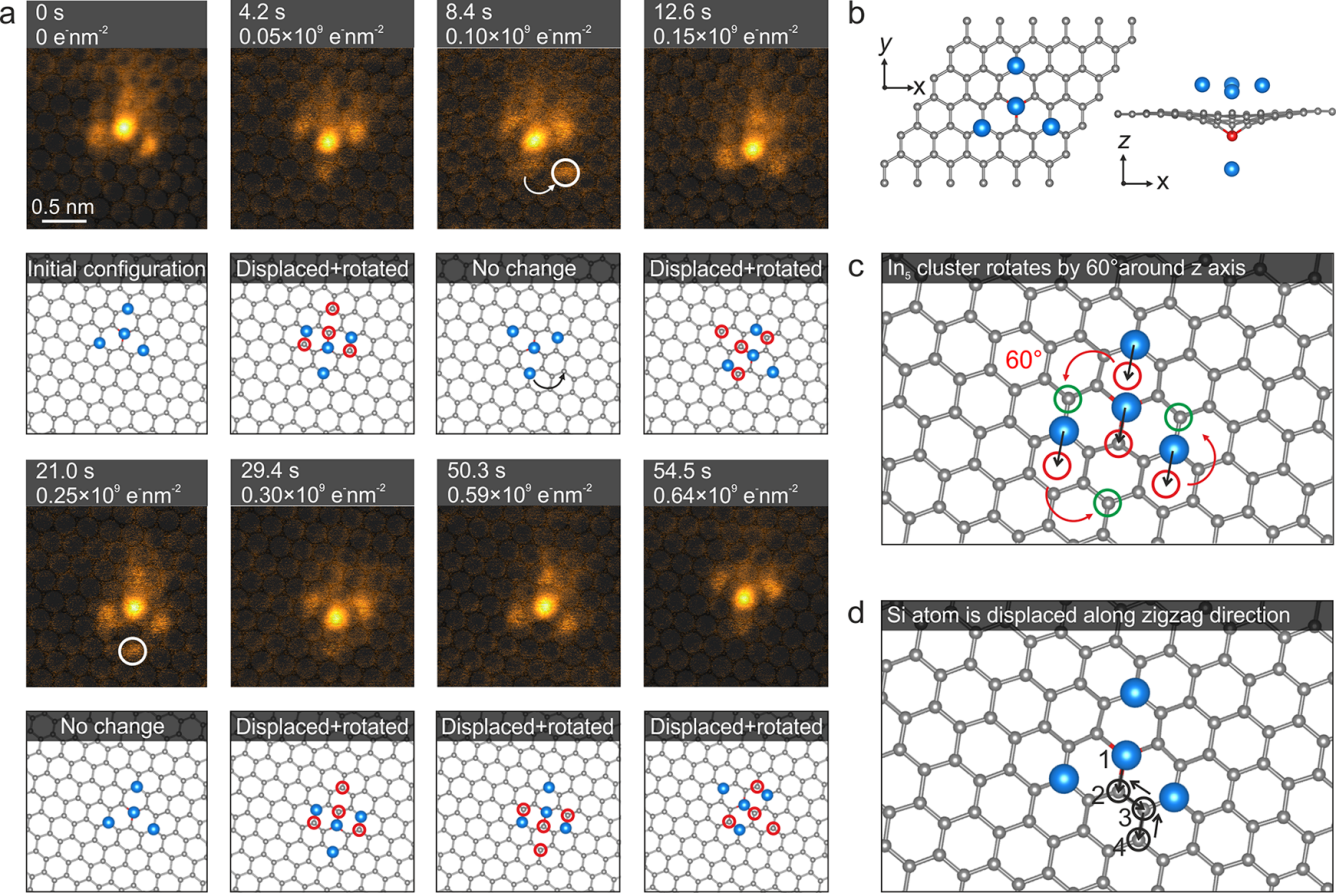
Dynamics of C-centered threefold symmetric In_5_ cluster under electron irradiation. (a) MAADF-STEM image sequence of the cluster acquired at different electron irradiation doses and the corresponding DFT-relaxed models. A semitransparent DFT model is superimposed on all images. The red circles represent the previous locations of In atoms before the cluster displaces and rotates. (b) DFT-relaxed model of C-centered In_5_. (c) DFT-relaxed model showing how the cluster rotates after displacement of the Si atom to a neighboring C site. The red circles indicate the position of In atoms after migration of cluster by half a lattice vector (see black arrows). The green circles show the position of three In atoms surrounding the center after the rotation of the cluster by 60°. (d) Same model as in (c), showing the initial configuration of the In_5_ cluster and the sites that the cluster occupies under electron irradiation. First, the center of the cluster moves step by step from position 1 to 4 (see black circles), and then it migrates back from position 4 to 2. The black arrows show the direction of movement. The experimental images are in false color and Wiener filtered (raw images are shown in SI Figure S12).

When irradiated by the e-beam at higher dose rates (smaller fields of view), we not only observe cluster formation and structural dynamics *within* the In clusters (Figures 2, 4, 5) but for some also translation/migration in their entirety along the graphene lattice: While the hexagon-centered In_3_ clusters are not observed to change their position during e-beam irradiation (see SI Video 3), we show in Figure 6a the dynamics of a C-centered In_5_ threefold symmetric In cluster during irradiation (see also SI Video 7). From its initial position, the cluster moves after 4.2 s (∼0.05 × 10^9^ e^–^ nm^–2^) with respect to the graphene lattice. The movement of the cluster is due to the migration of the Si impurity to a neighboring C site along the zigzag direction of graphene, presumably via the same bond inversion mechanism as Si impurity manipulation.^20^ The initial positions of the In atoms are marked by red circles in the DFT-relaxed model corresponding to the structure acquired at 4.2 s (∼0.05 × 10^9^ e^–^ nm^–2^). (Note that the Si atom is not visible in the model due to the In atom located on top of it.) The change in the location of the Si changes the alignment of the In atoms with respect to the graphene lattice, and thus the entire cluster migrates to preserve its bonding. The movement of the cluster continues after 8.4 s (∼0.10 × 10^9^ e^–^ nm^–2^). In the HAADF image, an additional In contrast appears below the cluster (same effect is visible in the frame at 21.0 s at a dose of ∼0.25 × 10^9^ e^–^ nm^–2^). We believe this to be because the cluster rotated before the scan was finished, which suggested acquiring STEM images at a higher scan speed. Although we were not able to resolve the rotation direction of the cluster, additional In contrast does appear on the HAADF image at 8.4 s (∼0.10 × 10^9^ e^–^ nm^–2^) suggesting that the cluster rotates 60° anticlockwise around the center (see also SI Figure S12). Until the last image frame acquired after 54.5 s (∼0.64 × 10^9^ e^–^ nm^–2^), the Si atom and the cluster have jumped five times along the zigzag direction of the graphene lattice (see path in Figure 6d). Although beyond the scope of this work, this observation sequence suggests that not only single atoms^18^ but also more complex heteroatomic structural assemblies could be positioned on the graphene lattice by electron-beam manipulation.

Finally, large-supercell semilocal DFT calculations^48^ and their resulting atom-decomposed densities of states (SI Figures S13–S14) indicate that the Si–In structures observed here on graphene are all approximately one- or two-electron deficient with a large local charge polarization and, as such, may be promising candidates for application screening, e.g., for single-site catalysis.^4^ Notably in this context, the rational anchoring approach presented here for In single atoms and In nanoclusters via Si anchors in the graphene lattice may also be extendable to other anchored metals as well as other solid supports including widely used carbon supports in heterogeneous catalysis. While our work here demonstrates the viability of self-assembly of Si-anchored In single atoms and nanoclusters on graphene under UHV conditions with stability at room temperature, the presented In–Si-graphene structures still need to prove their usability for actual catalysis or plasmonics applications. Such application screening will require, e.g., improving the selectivity of the preparation route toward few-atom structures (i.e., reducing the number of remaining larger In nanoparticles, see Figure 1b and SI Figure S1a) and assessing the stability, possible phase transformations, and activity of the In–Si-graphene structures under relevant, e.g., oxidative or reductive, reaction conditions in future *operando* type work.

## Conclusion

We report the self-assembly and anchoring of single In atoms and few-atom In clusters onto substitutional Si impurity atoms in suspended monolayer graphene membranes. A variety of structure types that are stable at room temperature are found from our facile fabrication route without the requirement for e-beam induced materials modification. Most frequently observed structures are hexagon-centered fourfold symmetric In_6_ clusters, single In atoms anchored on Si, and hexagon-centered threefold symmetric In_3_ clusters. Notably, the original coordination of the Si determines the atomic arrangements of the In structures: While single In atoms and threefold symmetric In clusters form on threefold coordinated Si impurities, fourfold symmetric clusters are found on fourfold coordinated Si impurities. Due to energy transfer from the scanning e-beam, in higher-dose-rate close-up imaging we observe *in situ* the formation, structural changes, and translation dynamics of the Si-anchored In structures on graphene: The hexagon-centered In_6_ fourfold symmetric clusters transform into three different structures during e-beam irradiation, including In chains and dimers. Unlike the fourfold symmetric clusters, the C-centered In_5_ threefold symmetric clusters can move under e-beam irradiation along the zigzag direction of graphene lattice and also transform to single In atoms anchored on the Si. The observed Si-anchored In structures on graphene are promising for future application screening in, e.g., catalysis. Combined, our atomic-scale observations provide a materials system and rational framework toward the controlled self-assembly and heteroatomic anchoring of single metal atoms and few-atom clusters on graphene.

## Methods

### STEM and EELS Measurements

STEM images were acquired with a Nion UltraSTEM100 operated at a 60 kV accelerating voltage in UHV (∼10^–9^ mbar) using concurrent high-angle annular dark field (HAADF) and medium-angle annular dark field (MAADF) detectors with collection angles of 80–300 mrad and 60–80 mrad, respectively. The EELS experiments were carried out by a Gatan PEELS 666 spectrometer retrofitted with an Andor iXon 897 electron-multiplying charge-coupled device camera. The energy dispersion, the beam current, and the EELS collection semiangle were 0.5–1 eV per channel, 30 pA, and 35 mrad, respectively.^49^ The STEM is equipped with a custom-made sample loading and transfer system to enable direct transfer of samples from various preparation chambers into the STEM without exposure to ambient.^50,51^

### *In Situ* Laser Cleaning

A tunable 6 W diode laser (445 nm, Lasertack GmbH) was used to clean the graphene surfaces. Laser irradiation of the sample held in a transfer arm was performed through a viewport in both STEM and UHV sample preparation chambers. In the experiments, the laser was operated at 10% duty cycle reducing the laser power to 600 mW, which does not induce structural damage in graphene but is sufficient for cleaning.^38,39^ Sample irradiation time was 2 ms.

### *In Situ* In Deposition

The *in situ* evaporation of In was achieved using a custom-built preparation chamber (base pressure ∼10^–9^ mbar) coupled to the STEM. The evaporation source was a Knudsen cell with In pellets (99.99% purity, Kurt J. Lesker), which were heated to 700 °C. The resulting In flux was then directed at the graphene sample, which was not intentionally heated. Nominally deposited In thickness was monitored using a quartz microbalance and kept to ∼10 nm. Substrates used for In deposition were commercial CVD graphene (Graphenea Inc.) suspended on perforated silicon nitride grids (Ted Pella Inc.). Subsequent to *in situ* In evaporation, a second *in situ* laser cleaning step was applied to the sample.

### STEM Image Simulations

HAADF and MAADF image simulations were carried out using the QSTEM software with parameters corresponding to the experiments:^52^ chromatic aberration coefficient of 1 mm, a spherical aberration coefficient of 1 μm, and energy spread of 0.48 eV. HAADF and MAADF detector angle ranges are set to the experimental range of 80–300 mrad and 60–80 mrad, respectively.

### Image Processing

ADF images were processed to reduce noise and increase contrast via double Gaussian filtering,^28^ in some cases after applying Wiener filtering. The parameters used for double Gaussian filtering are σ1 = 0.25, σ2 = 0.20, weight = 0.3 (for Figure 1c,e,f); σ1 = 0.25, σ2 = 0.22, weight = 0.25 (for Figure 2a); σ1 = 0.25, σ2 = 0.22, weight = 0.25 (for Figure 2e–g); σ1 = 0.36, σ2 = 0.26, weight = 0.20 (for Figure 3a); σ1 = 0.32, σ2 = 0.25, weight = 0.15 (for Figure 3b); and σ1 = 0.35, σ2 = 0.26, weight = 0.30 (for Figure 4). For the images in Figure 1, Figure 5, and Figure 6, the low-pass Wiener filter^53^ was applied. In addition, we used false coloring with the ImageJ lookup table ”Orange Hot”.

### DFT Simulations

Density functional theory (DFT) simulations were carried out using the grid-based projector-augmented wave (GPAW) software package^54^ to study the properties of the supercells of monolayer graphene with Si impurities and adsorbed In clusters. The atomic structures were relaxed with the PBE functional and periodic boundary conditions (with >10 Å of vacuum in the perpendicular direction between the images) in the LCAO mode^54^ with the grid spacing of 0.2 Å and a 5 × 5 × 1 **k**-point mesh so that maximum forces were <0.02 eV Å^–1^.^55^ For details of large-supercell density of states calculations, Wannier-function decomposed in order to assess charge transferability and performed using the linear-scaling package ONETEP;^48^ see SI Figures S13–S14.

## Supporting Information Available

Additional STEM and EELS data and additional DFT data and corresponding discussions (PDF)
Video 1: Raw STEM data (AVI)
Video 2: Raw STEM data (AVI)
Video 3: Raw STEM data (AVI)
Video 4: Raw STEM data (AVI)
Video 5: Raw STEM data (AVI)
Video 6: Raw STEM data (AVI)
Video 7: Raw STEM data (AVI)

## Supplementary Material

nn1c03535_si_001.pdf

nn1c03535_si_002.avi

nn1c03535_si_003.avi

nn1c03535_si_004.avi

nn1c03535_si_005.avi

nn1c03535_si_006.avi

nn1c03535_si_007.avi

nn1c03535_si_008.avi
