# Supplementary material for: Single Indium Atoms and Few-Atom Indium Clusters Anchored onto Graphene via Silicon Heteroatoms
Source: ACS Nano. 2021 Aug 19;15(9):14373–83. doi: 10.1021/acsnano.1c03535 (PMC8482752; doi:10.1021/acsnano.1c03535)
Supplement: Supplementary file 1 — nn1c03535_si_001.pdf [file nn1c03535_si_001.pdf]

Supporting Information:

Single Indium Atoms and Few-Atom Indium Clusters Anchored onto Graphene *via* Silicon Heteroatoms

Kenan Elibol,[1,2,3] Clemens Mangler,[1] David D. O'Regan,[2,4] Kimmo Mustonen,[1] Dominik Eder,[5] Jannik C. Meyer,[1,6] Jani Kotakoski,[1] Richard G. Hobbs,[2,3] Toma Susi,[1,] Bernhard C. Bayer[1,5,*]*

[1]University of Vienna, Faculty of Physics, Boltzmanngasse 5, A-1090, Vienna, Austria

[2]Centre for Research on Adaptive Nanostructures and Nanodevices (CRANN) and the SFI Advanced Materials and Bio-Engineering Research Centre (AMBER), Dublin 2, Ireland

[3]School of Chemistry, Trinity College Dublin, The University of Dublin, Dublin 2, Ireland

[4]School of Physics, Trinity College Dublin, The University of Dublin, Dublin 2, Ireland

[5]Institute of Materials Chemistry, Vienna University of Technology (TU Wien), Getreidemarkt 9/165, A-1060 Vienna, Austria

[6]Institute for Applied Physics, University of Tübingen, Auf der Morgenstelle 10, 72076 Tübingen, Germany

*Corresponding authors: bernhard.bayer-skoff@tuwien.ac.at, toma.susi@univie.ac.at

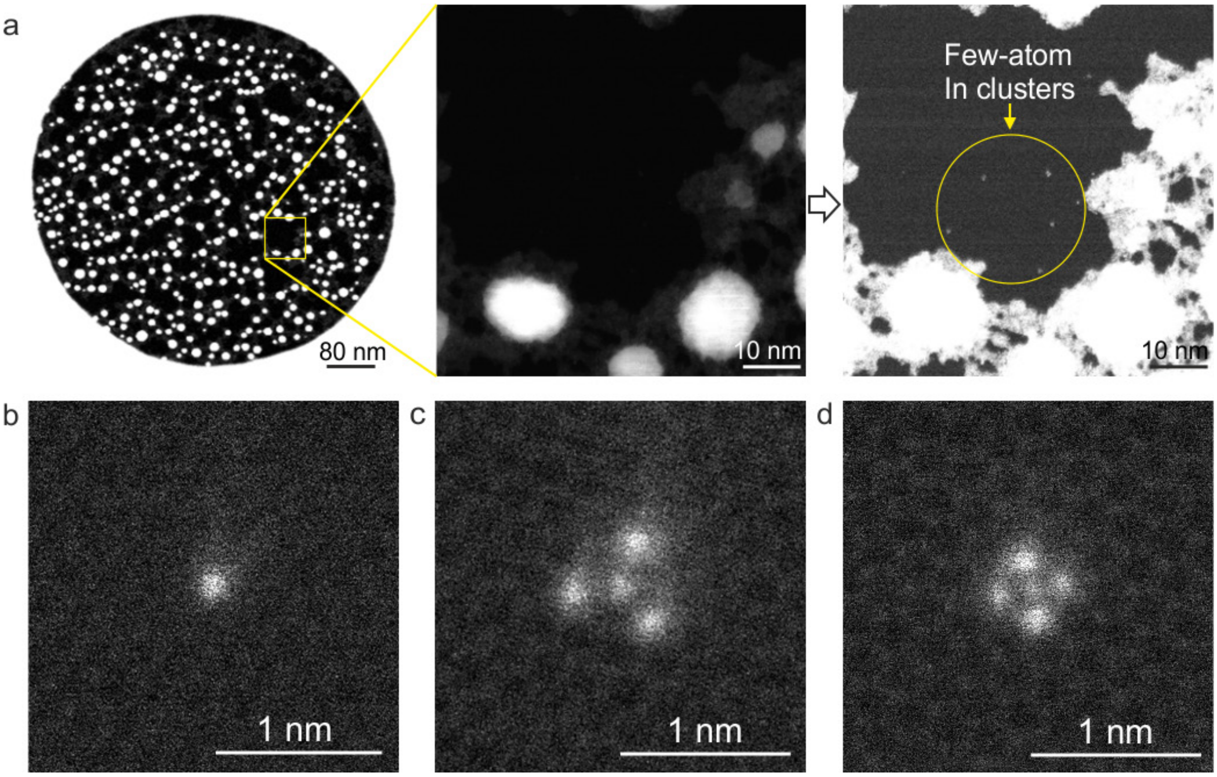

Supporting Figure S1. (a) Overview MAADF-STEM images of In clusters and surrounding In nanoparticles. (b-d) Raw MAADF-STEM images of a single In, 3-fold and 4-fold symmetric In clusters shown in Figure 1 in the main text.

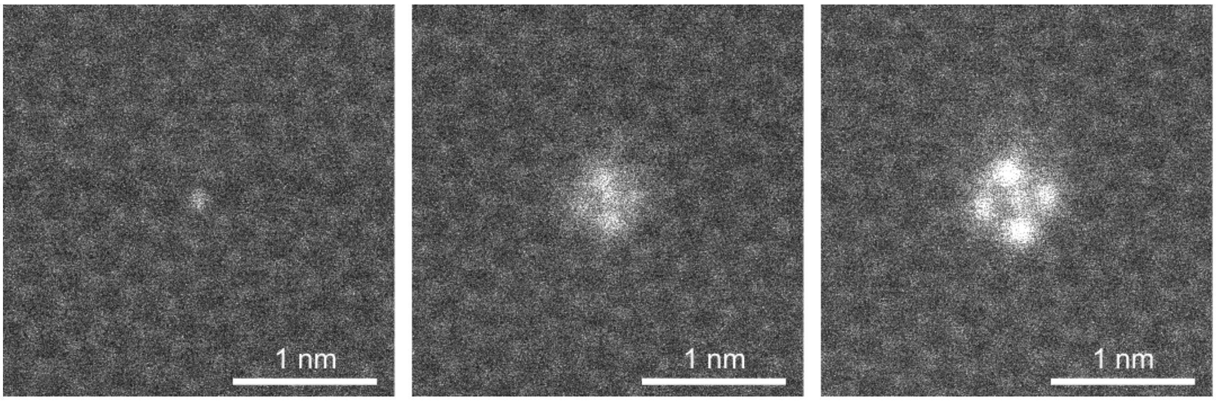

Supporting Figure S2. Raw MAADF-STEM images of the structures shown in Figure 2 in the main text.

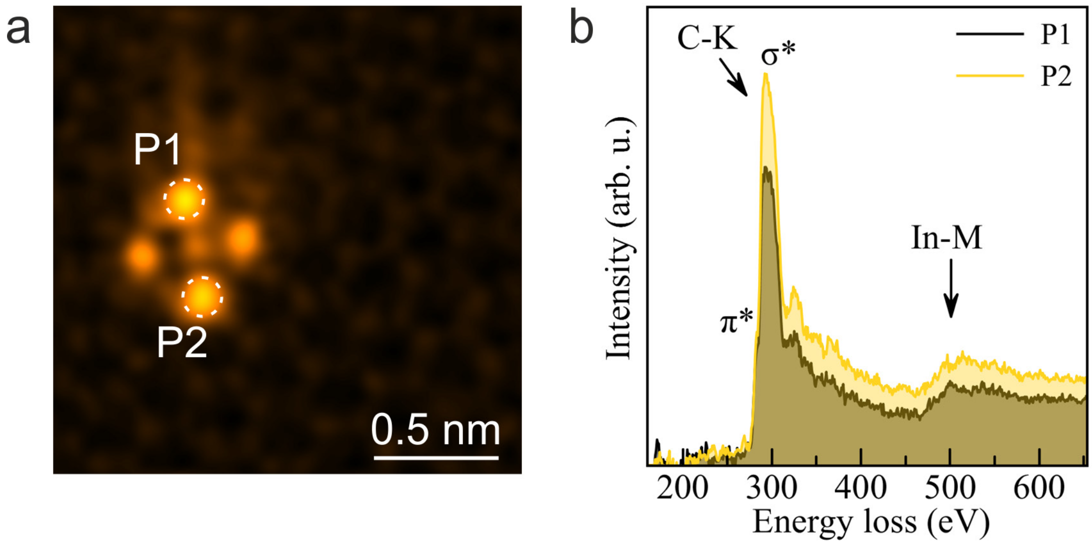

Supporting Figure S3. (a) HAADF-STEM image of a 4-fold symmetric cluster (replotted from Figure 2a) and (b) EEL point spectra acquired over the brighter atoms (P1 and P2) marked by white dashed circles on panel (a). The energy dispersion is 1 eV/px and the background has been subtracted by power-law fitting.

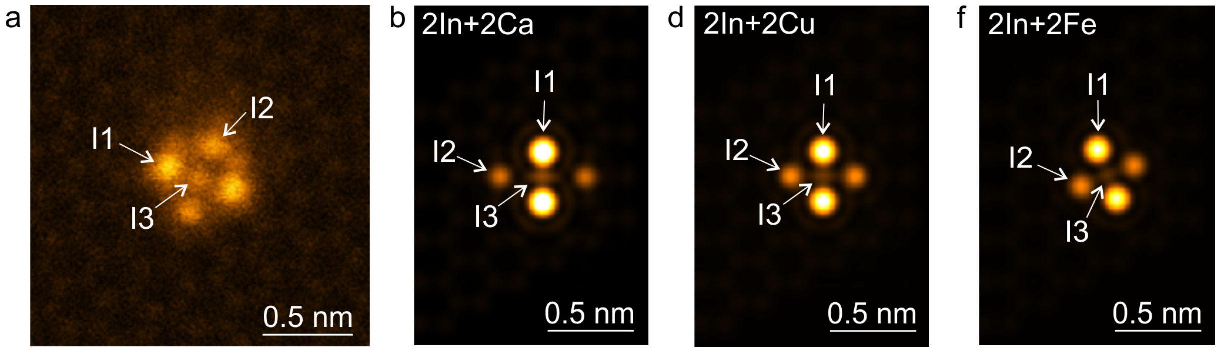

h

	Exp.	2In+ 2Ca	2In+ 2Cu	2In+ 2Fe
I1/I2	1.35	5.69	2.98	3.43
I1/I3	1.68	7.35	10.18	8.61
I2/I3	1.24	1.29	3.41	2.51

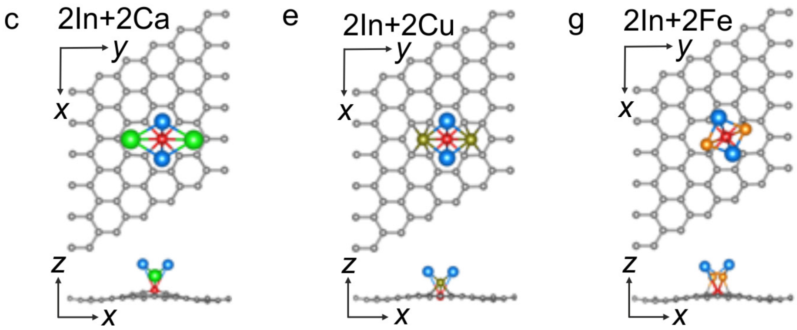

Supporting Figure S4. (a) HAADF-STEM image of the hexagon-centered 4-fold symmetric In cluster shown in Figure 3a. (b) Simulated HAADF images and corresponding DFT-relaxed models for the structures consisting of (b,c) 2 In + 2 Ca, (d,e) 2 In + 2 Fe and (f,g) 2 In + 2 Cu, respectively. (h) A table showing the intensity ratios measured on the atoms marked in the experimental and simulated HAADF images.

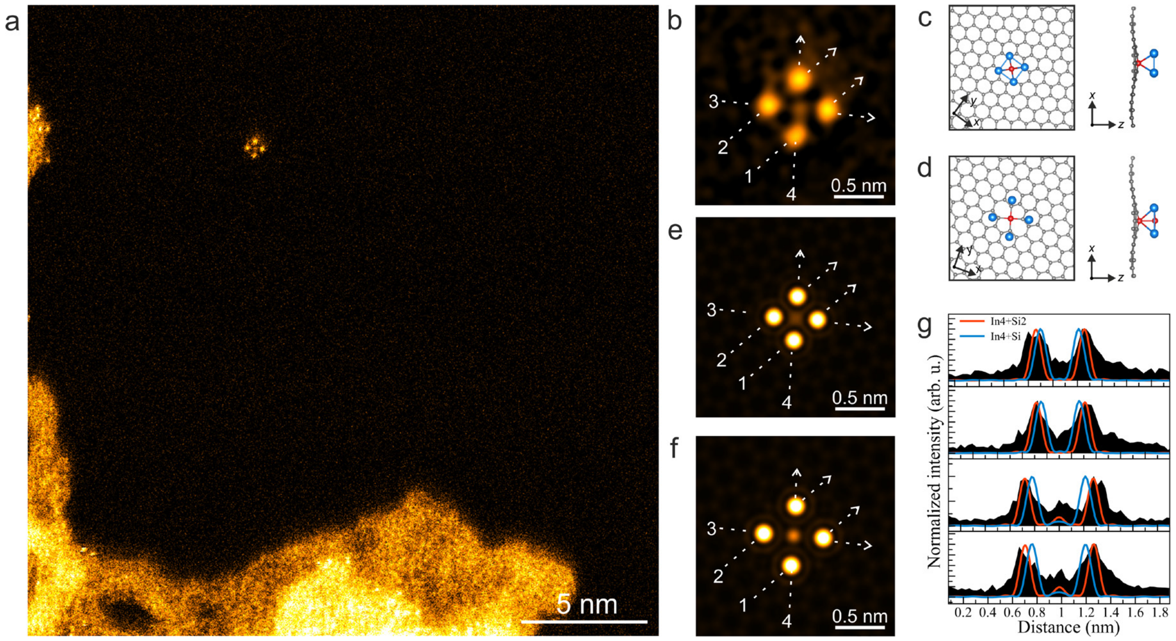

Supporting Figure S5. (a) HAADF-STEM image of a 4-fold In cluster. (b) Close-up and double Gaussian filtered HAADF-STEM image of the cluster in panel a. (c) DFT-relaxed model consisting of (c) four In atoms on a 4-fold coordinated Si and (d) four In atoms and a Si atom on the 4-fold coordinated Si. (e,f) Simulated HAADF images of the models in panels c and d, respectively. (g) Intensity profiles over the white dashed lines on panels b, e and f.

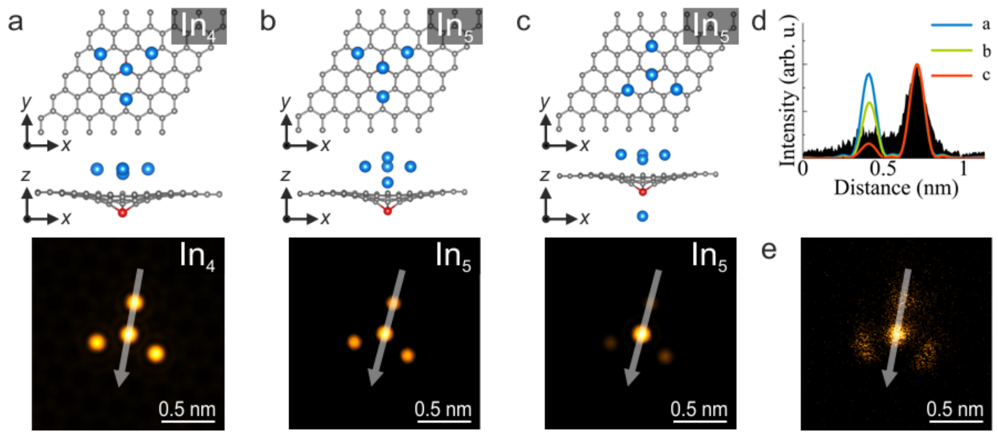

Supporting Figure S6. Plan and side views of a DFT-relaxed models and corresponding simulated HAADF images showing 3-fold symmetric (a) In_4, (b) In_5 (two In atoms on top of each other at the center of cluster) and (c) In_5 (one In atom above and one under Si atom) clusters. (d) Intensity profiles recorded along semi-transparent white lines over simulated (a,b,c) and experimental (e) HAADF images. (e) Raw HAADF-STEM image of a 3-fold symmetric In_5 cluster.

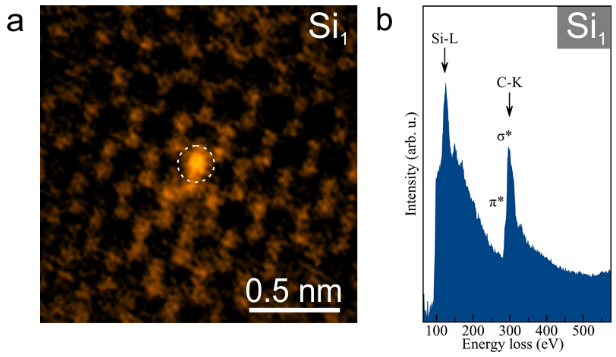

Supporting Figure S7. (a) HAADF-STEM image of Si_1 in graphene lattice, corresponding to Figure 5j. (b) EEL spectrum acquired over the brightest atom in panel (a).

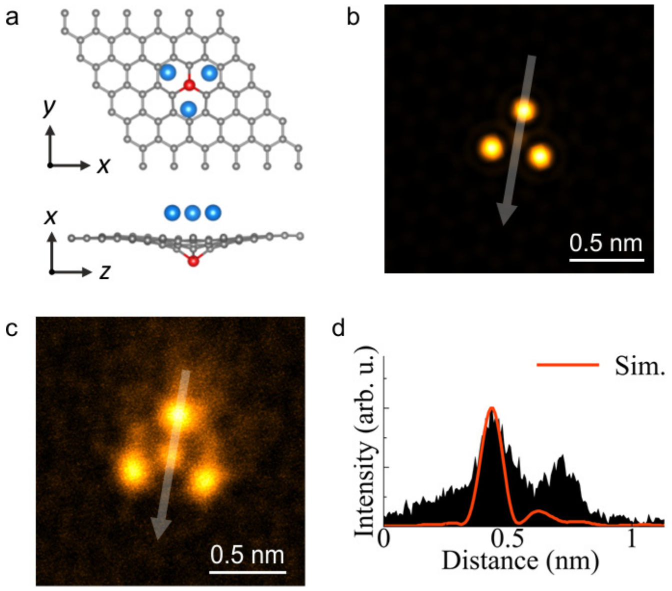

Supporting Figure S8. (a) Plan and side views of a DFT-relaxed model showing 3-fold symmetric In cluster without an extra In or Si atom at the center of the cluster and (b) its corresponding simulated HAADF image. (c) HAADF-STEM image of hexagon-centered 3-fold symmetric In_3 cluster. (d) Intensity profiles recorded along semi-transparent white lines over simulated (b) and experimental (c) HAADF images.

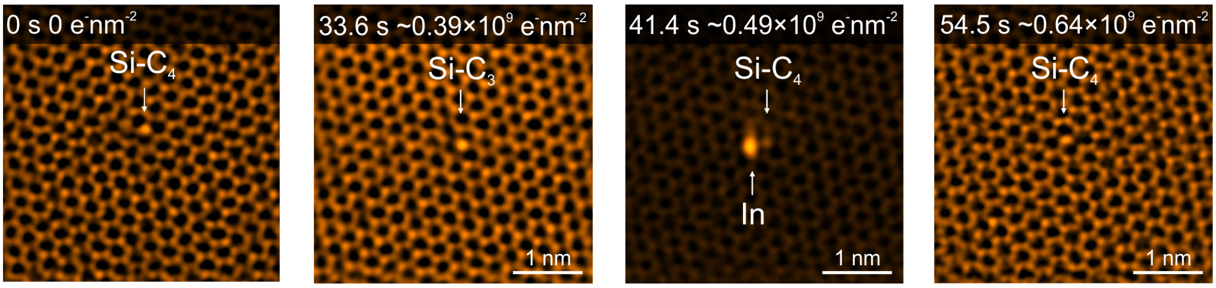

Supporting Figure S9. MAADF-STEM image sequence showing a Si atom switching between 3-fold and 4-fold coordinated configurations under electron irradiation. The image acquired after 41.4 s shows an In atom briefly trapped by a 4-fold coordinated Si. Images are double Gaussian filtered after Wiener filtering.

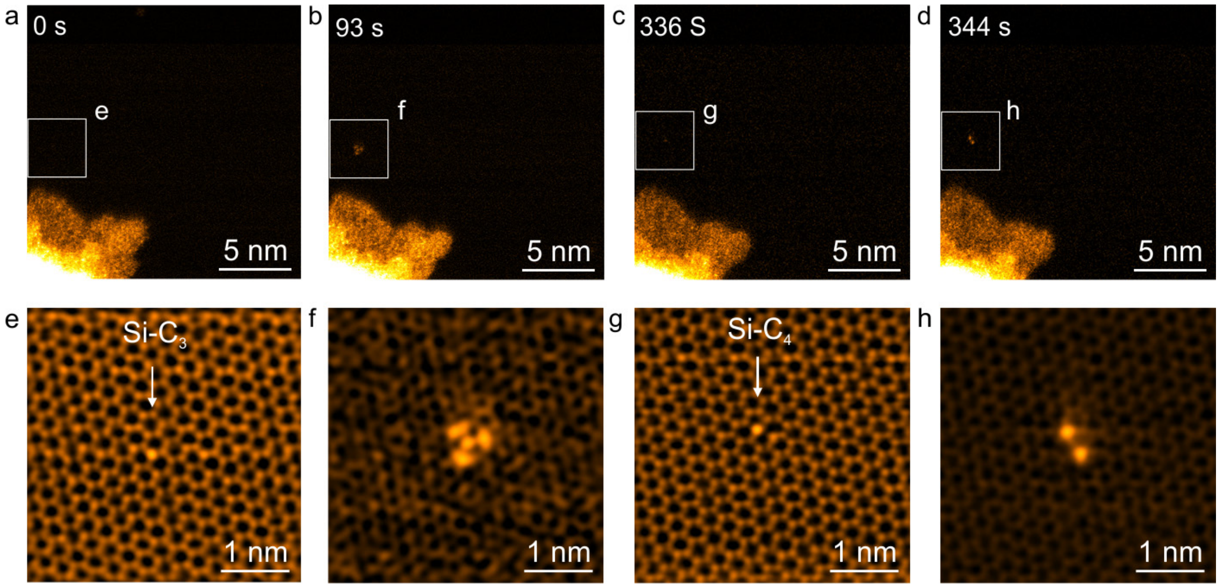

Supporting Figure S10. (a-d) Large-area MAADF-STEM images. (e-f) Close up MAADF-STEM images of the areas indicated by the white frames on the images in panels a-d. The electron dose rates are 0.53×10^6 $e^-nm^{-2}s^{-1}$ (a-d) and 11.72×10^6 $e^-nm^{-2}s^{-1}$ (e-h). Images in panels a-d display raw data in false color whereas images in panels e-h are double Gaussian filtered after Wiener filtering.

DFT total energy calculations for evaluating In cluster stability

In an attempt to understand why we experimentally observe very specific In clusters and not others, we performed extensive DFT structure optimizations and total energy comparisons for the experimentally observed clusters as well as for hypothetical structures with added In atoms. Notably, for the observed structures, we find no clear correlation of DFT-derived formation energy and experimental areal observation density (Supporting Table 1), pointing to the role of kinetic effects in structure formation. For testing the energy change from further In addition to the experimentally observed structures, one additional In atom was added manually at manually selected positions around the experimentally observed structures. Both E per In and ΔE for +In indicate that adding a further In atom to the experimentally observed structures is energetically favoured (Supporting Table 2). Thus DFT does not explain why our experimentally observed structures are limited to single In and few-atom In nanoclusters (2-6 atoms) instead of larger cluster formation. This also suggests kinetic effects to play a role in the formation of the observed structures, see also main text.

Supporting Table 1. DFT-derived total energies (E_{tot}), number (#) of C, Si and In atoms, chemical potential for each element (Chem. pot.), formation energy (Form. E), energy (E) per In (comparing the energy of each structure to the corresponding 4-fold/3-fold Si-doped graphene model, divided by the number of In atoms in the structure), reference to the corresponding atomic model in the main text, and the experimentally observed areal observation density (ρ_{obs}) per 1000 nm^{-2} from the main text for all salient, experimentally observed structures.

System	E_{tot} (eV)	# C	# Si	# In	Chem. pot. (eV)	Form. E (eV)	E per In (eV)	Atomic model	ρ_{obs} (/1000 nm^{-2})
graphene	-640.786	72	0	0	-8.90			graphene lattice of 72 atoms	
bulk silicon	-10.601	0	2	0	-5.30				
bulk indium	-8.349	0	0	3	-2.78				
3-fold coordinated Si in graphene	-633.264	71	1	0		3.92			0.89
4-fold coordinated Si in graphene	-623.586	70	1	0		4.70			0.82
hexagon-centered 4-fold In_6	-635.000	70	1	6		9.99	-1.90	Fig. 3c	0.37
C-centered 4-fold In_4	-630.664	70	1	4		8.75	-1.77	Fig. 3d	0.20
In_6 chains	-634.195	70	1	6		10.79	-1.77	Fig. 4c, 16.8 s	0.07
In_2 dimers	-627.586	70	1	2		6.27	-2.00	Fig. 4c, 100.7 s	0.12
C-centered 3-fold symmetric In_5	-640.643	71	1	5		10.46	-1.48	Fig. 5b	0.15
single In atom anchored on Si	-634.590	71	1	1		5.38	-1.33	Fig 5e	0.32
hexagon-centered In_3 3-fold symmetric clusters (with extra Si)	-641.489	71	2	3		9.35		Fig. 5h	0.22

Supporting Table 2. DFT-derived total energies (E_{tot}), number (#) of C, Si and In atoms, formation energy (Form. E), energy (E) per In (comparing the energy of each structure to the corresponding 4-fold/3-fold-doped graphene model, divided by the number of In atoms in the structure), change in energy after structural optimization (ΔE for +In) when adding an In atom at selected positions to the stable structure with one fewer In atom, sketch of the relaxed atomic structure and, for selected experimentally observed structures (whose values are shown in bold font), reference to the corresponding atomic model in the main text.

**System**	**E_{tot} (eV)**	**# C**	**# Si**	**# In**	**Form. E (eV)**	**E per In (eV)**	**ΔE for +In (eV)**	**Atomic model**
**In_2 dimers**	**-627.586**	**70**	**1**	**2**	**6.27**	**-2.00**	**-2.00**	Fig. 4c, 100.7 s 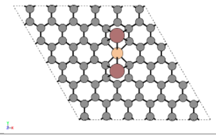
+ 1 top In (position 1)	-629.108	70	1	3	7.53	-1.84	-1.52	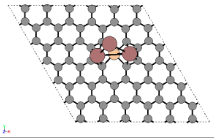
+1 top In (position 2)	-629.115	70	1	3	7.52	-1.84	-1.53	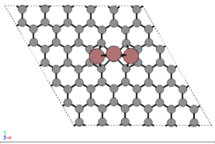
+ 1 top/end In (position 3)	-629.420	70	1	3	7.22	-1.94	-1.83	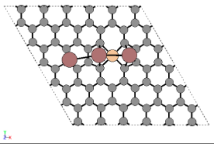

System	E_{tot} (eV)	# C	# Si	# In	Form. E (eV)	E per In (eV)	ΔE for +In (eV)	Atomic model
**In_6 chains**	**-634.195**	**70**	**1**	**6**	**10.79**	**-1.77**	**-2.09**	Fig. 4c, 16.8 s 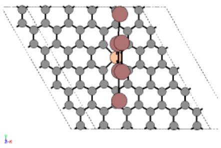
+ 1 central In	-636.228	70	1	7	11.54	-1.81	-2.03	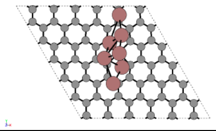
**hexagon-centered 4-fold In_4**	**-630.664**	**70**	**1**	**4**	**8.75**	**-1.77**	**-1.77**	Fig. 3d 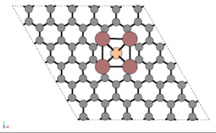
+ 1 top In (position 1)	-632.434	70	1	5	9.77	-1.77	-1.77	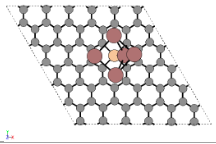
+ 1 top In (position 2)	-632.597	70	1	5	9.60	-1.80	-1.93	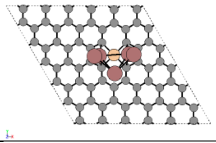
+ 1 top In (position 3)	-633.041	70	1	5	9.16	-1.89	-2.38	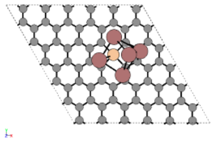
**hexagon-centered 4-fold In_6**	**-635.000**	**70**	**1**	**6**	**9.99**	**-1.90**	**-2.17**	Fig. 3c 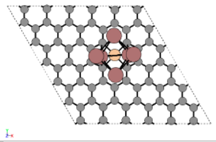
+ 1 bottom In	-636.887	70	1	7	10.88	-1.90	-1.89	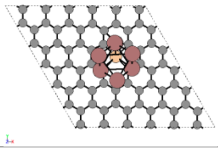
+ 1 top In	-637.337	70	1	7	10.43	-1.96	-2.34	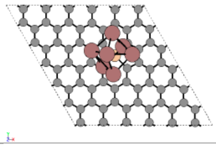
+ 1 side In	-637.410	70	1	7	10.36	-1.97	-2.41	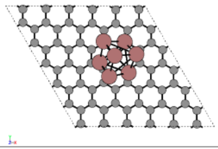
+ 1 central In	-637.560	70	1	7	10.21	-2.00	-2.56	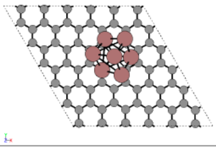

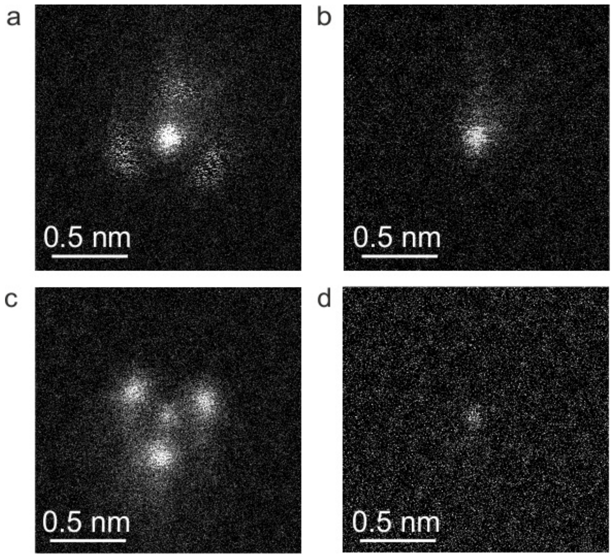

Supporting Figure S11. Raw HAADF-STEM images of the structures shown in Figure 5 in the main text. (a) C-centered 3-fold symmetric In_5 cluster. (b) Single In atom anchored onto the Si impurity. (c) Hexagon-centered 3-fold symmetric In_3 cluster. (d) 3-fold coordinated Si atom in graphene.

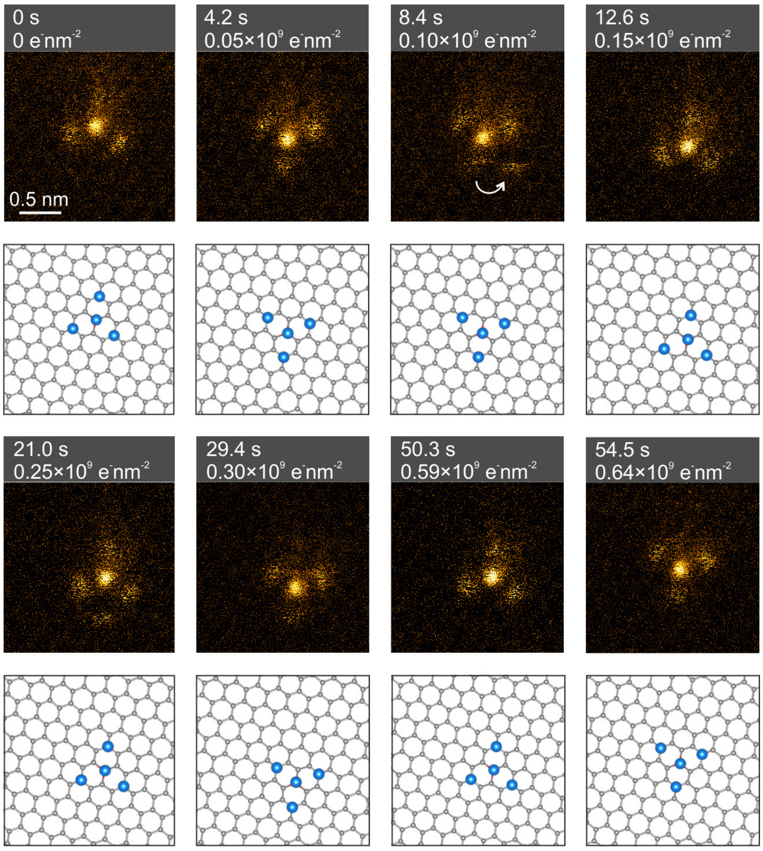

Supporting Figure S12. MAADF-STEM image sequence of C-centered 3-fold symmetric In_5 cluster shown in Figure 6 in the main text.

Electronic structure assessment by means of large-scale DFT

In Supporting Figures S13-S14 we show the results of large-scale DFT calculations performed to analyze the electronic properties of the observed Si-anchored In structures on graphene in good isolation from their periodic images in the simulation. These calculations were run particularly to better understand the effect of In atom count on their spectral properties, and to estimate their relevance for applications in, *e.g.*, single-site catalysis.

Specifically, we performed geometry relaxation and atomic population analysis using large-scale Kohn-Sham density-functional theory (DFT)[1] on 18 × 18 (~650 atom) supercells. We applied the linear-scaling DFT code ONETEP,[2–4] which uses a minimal basis of spatially-localized functions, called nonorthogonal generalized Wannier functions (NGWFs), to expand the Kohn-Sham orbitals. Accuracy equivalent to that of a plane-wave pseudopotential code was attained by variationally optimizing those Wannier functions *in situ*, separately at each geometry optimization step, in order to minimize the total energy and to refine the Hellmann-Feynman forces, including Pulay corrections,[5] used for Born-Oppenheimer geometry optimization. An out-of-plane lattice constant of 25 Å was used to separate graphene sheets from their periodic images, and an equivalent plane-wave kinetic energy cutoff of ~1020 eV was used. A common, 6.35 a_0 NGWF cutoff radius was applied, and no truncation of the density-matrix was applied. For each atom of a species, 4 NGWFs were allocated to C atoms, 4 to Si atoms, and 9 to In atoms, *i.e.* the outermost d-electrons were kept in the In valence. The PBE[6] semi-local generalized gradient exchange correlation functional was used, together with corresponding scalar-relativistic, norm-conserving pseudopotentials that we generated using the Opium code,[7] including soft non-linear core corrections. In geometry optimization, a total-energy convergence tolerance of 10^{-6} Ha/atom, force tolerance of 2×10^{-3} Ha/Bohr, and displacement tolerance of

5×10^{-3} Bohr were maintained over a convergence window of 4 geometry steps. A Gaussian smearing half-width of 0.1 eV was used in our species-decomposed Kohn-Sham density of states plots. NGWF-based Mulliken population analysis was used to partition the density, and density of states, per chemical species, and then we further divided each density of states by the number of atoms of each species to analyze how each atom contributes.

We find using Wannier-function based Mulliken atomic population analysis that for the single In atom (Supporting Figure S13a,d), a charge of approximately 1.2 e is transferred from the graphene. This is shared between the Si atom (+0.8 e) and the In atom (+0.4 e each), with the three neighboring C atoms receiving -0.4 e and the remainder being delocalized. The optimized bond lengths in the cluster are 1.82 Å (C-Si), and 2.87 Å (Si-In). The per-atom Kohn-Sham density of states in this cluster is dominated by near-degenerate In states distributed around the Fermi level, with further sharp In peaks spread over higher energies. This suggests that the cluster may serve as an amphoteric but more probably acceptor-like binding site, and even as a catalytic site, for small molecules.[8,9]

Referring next to the two In_2 dimer configurations (Supporting Figure S13b,d for hexagon-centered and 13c,f for pentagon-centered), a larger charge of approximately 2.1 e is transferred from the graphene. This is shared between the Si atom (+0.9 e) and almost equally between the two In atoms (+0.6 e each), primarily donated by the 4 nearby C atoms (-0.4 e each). The optimized bond lengths in the hexagonal-centered cluster are, on average 1.89 Å (C-Si), 3.04 Å (Si-In), 4.01 Å (In-In). The optimized bond lengths in the pentagon-centered cluster are, on average 1.90 Å (C-Si), 2.94 Å (Si-In), 3.65 Å (In-In). The per-atom Kohn-Sham density of states for both of these clusters is characterized by a half-filled four-fold-

degenerate peak at the Fermi level, of predominantly In character. A dense group of In levels is spread over higher energies.

In the In_4 cluster (Supporting Figure S14a-c), a charge of approximately 1.9 e is transferred from the graphene. This is shared between the Si atom (+0.8 e) and almost equally between the four In atoms (+0.3 e each), and is primarily donated by 4 nearby C atoms (-0.4 e each). The optimized bond lengths in the cluster are, on average 1.95 Å (C-Si), 3.30 Å (Si-In), 2.75 Å (In-C), with the In-In bond lengths coming in pairs of length 3.07 Å and 3.14 Å. Amongst those studied, this cluster exhibits the most prominent partially-filled In peak at the Fermi level.

Finally, in the In_6 cluster (Supporting Figure S14d-f), approximately 1.7 e is transferred, but now the only significantly charged atoms are the Si (+0.8 e), the 2 lowest-lying In atoms adjacent to Si (+0.3 e), and the 4 C atoms bound to Si (-0.3 e). The cluster structure is more complex, comprising pairs of In atoms at three different altitudes with respect to the graphene surface, with the atoms in the first and third rows being aligned close to vertically with respect to each other, above the center of the 5-member C-Si ring. The second row sits above the center of the 6-member C-Si ring. The intra-row In-In bond length varies, from first to third row, as 3.24 Å, 4.33 Å, 4.14 Å. The other salient bond lengths are 1.92 Å (C-Si), 3.30 Å (Si-In), 2.91 Å (Si-In first row), 2.93 Å (In-In first row to third row). The first-row In atoms are sometimes closer to the C atoms in the 5-member C-Si ring than to the Si atom, at distances of approximately 2.8-2.9 Å. The per-atom Kohn-Sham density of states in this cluster again reveals a Fermi density dominated by In states, albeit not as sharply so as in the 4-atom case and, for a given energy, not equally among the In rows. Again, the charge-transfer to the cluster from the graphene serves to pin the bottom of the In 5p-like density of states to the

Fermi level, potentially providing overall a relatively spatially-localised but prominent conduit for further charge transfer and possible associated catalytic activity.[10–12]

On the basis of these calculations alone it is of course impossible to judge which of these clusters may provide the best activity and selectivity for a given chemical reaction, if any are indeed suitable for single-site catalysis. However, we can say that all of them should provide a strongly polarizing localized environment, and most likely act as electron acceptors, possibly amphoterically. A range of In-In and In-Si bond lengths are available across the clusters studied, and shorter bond lengths may suggest greater resistance to catalyst erosion and consumption. Overall, these clusters present an interesting avenue to extent the concept of the single-atom catalyst to more complex cluster geometries.

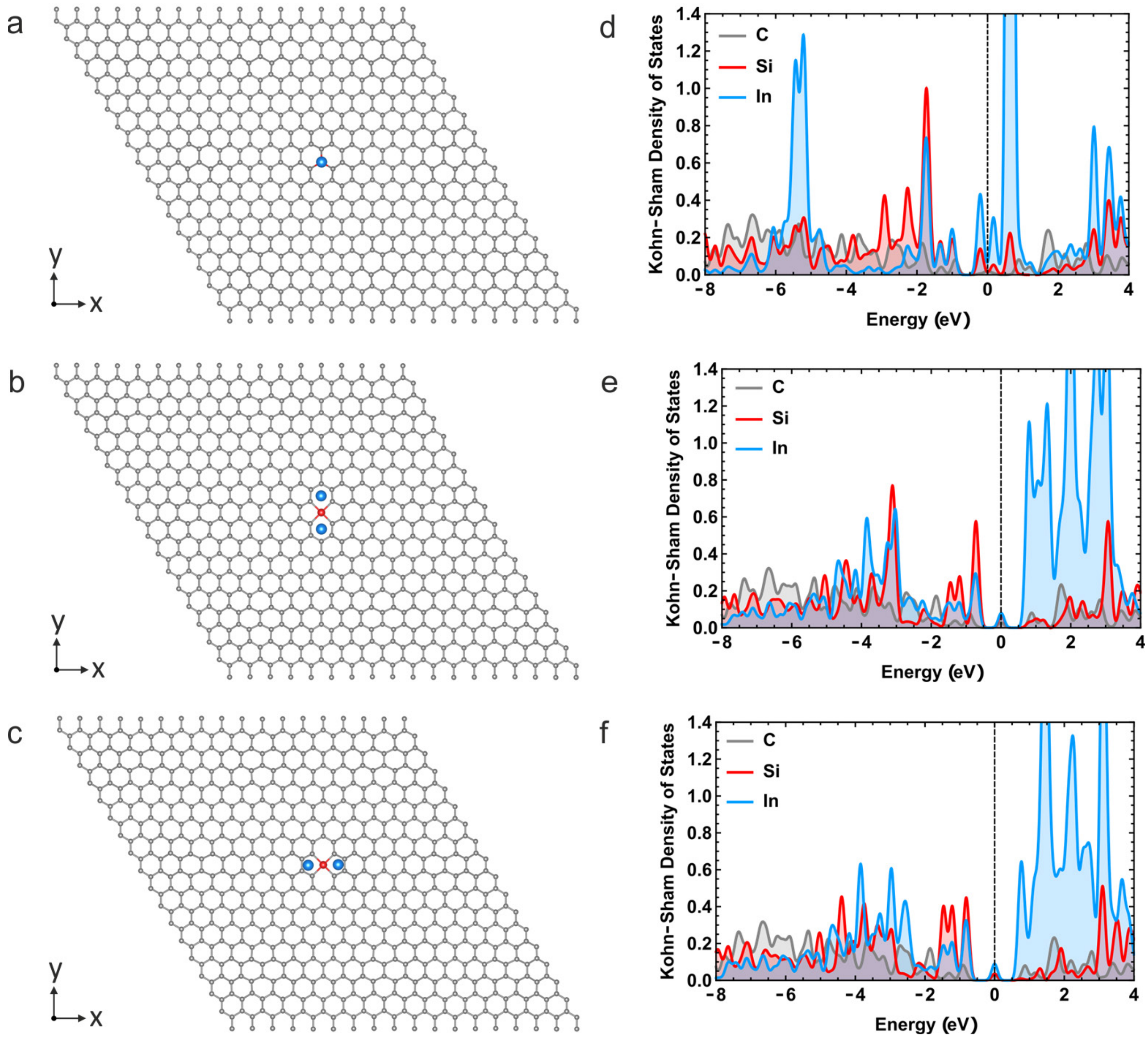

Supporting Figure S13. DFT-relaxed models showing 18 × 18 graphene cells containing (a) single In, (b) In_2 (hexagon-centered) and (c) In_2 (pentagon-centered) clusters. (d-f) Wannier-function based local density of Kohn-Sham states for single In atom, hexagon-centered In_2 dimer and pentagon-centered In_2 dimer, respectively, renormalized per atom.

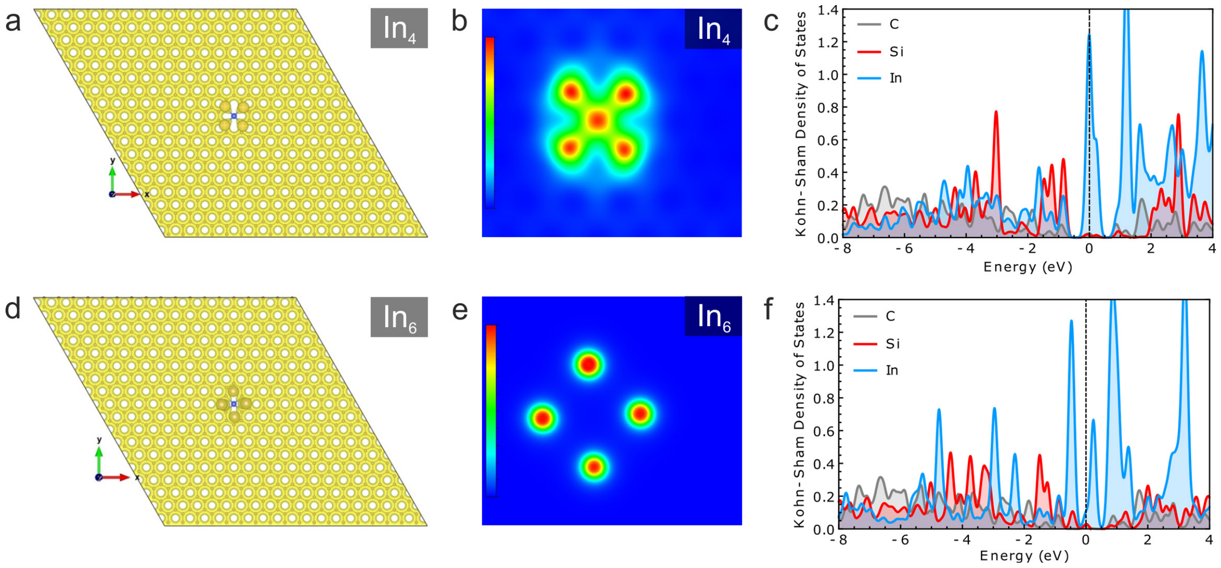

Supporting Figure S14. The electron density and Kohn−Sham density of states. (a,d) Isosurfaces of the calculated charge densities for 18 × 18 graphene cells containing 4-fold symmetric In_4 and In_6 clusters. (b,e) Contour plots showing zoomed-in planar cross-sections of the charge densities of 4-atom and 6-atom clusters, where the colour scale is set close to the minimum and maximum values on each plane. In the 4-atom case in (b), this cross-section is taken on a plane parallel to the graphene, equidistant between the Si atom and the 4 In atoms. In the 6-atom case in (e), the plane is instead set at an altitude above graphene that is equidistant between the second and third In rows. (c,f) Wannier-function based local density of Kohn-Sham states for 4-atom and 6-atom 4-fold symmetric clusters, respectively, renormalized per atom.

SUPPORTING REFERENCES

(1) Kohn, W.; Sham, L. J. Self-Consistent Equations Including Exchange and Correlation Effects. *Phys. Rev.* **1965**, *140*, A1133–A1138.

(2) Skylaris, C.-K.; Haynes, P. D.; Mostofi, A. A.; Payne, M. C. Introducing ONETEP: Linear-Scaling Density Functional Simulations on Parallel Computers. *J. Chem. Phys.* **2005**, *122*, 084119.

(3) Hine, N. D. M.; Robinson, M.; Haynes, P. D.; Skylaris, C.-K.; Payne, M. C.; Mostofi, A. A. Accurate Ionic Forces and Geometry Optimization in Linear-Scaling Density-Functional Theory with Local Orbitals. *Phys. Rev. B* **2011**, *83*, 195102.

(4) Prentice, J. C. A.; Aarons, J.; Womack, J. C.; Allen, A. E. A.; Andrinopoulos, L.; Anton, L.; Bell, R. A.; Bhandari, A.; Bramley, G. A.; Charlton, R. J.; Clements, R. J.; Cole, D. J.; Constantinescu, G.; Corsetti, F.; Dubois, S. M.-M.; Duff, K. K. B.; Escartín, J. M.; Greco, A.; Hill, Q.; Lee, L. P.; *et al.* The ONETEP Linear-Scaling Density Functional Theory Program. *J. Chem. Phys.* **2020**, *152*, 174111.

(5) Ruiz-Serrano, Á.; Hine, N. D. M.; Skylaris, C.-K. Pulay Forces from Localized Orbitals Optimized *in Situ* Using a Psinc Basis Set. *J. Chem. Phys.* **2012**, *136*, 234101.

(6) Perdew, J. P.; Burke, K.; Ernzerhof, M. Generalized Gradient Approximation Made Simple. *Phys. Rev. Lett.* **1996**, *77*, 3865–3868.

(7) Grinberg, I.; Ramer, N. J.; Rappe, A. M. Transferable Relativistic Dirac-Slater Pseudopotentials. *Phys. Rev. B* **2000**, *62*, 2311–2314.

(8) Mitchinson, A.; Finkelstein, J. Small-Molecule Catalysis. *Nature* **2008**, *455*, 303–303.

(9) Sudheeshkumar, V.; Sulaiman, K. O.; Scott, R. W. J. Activation of Atom-Precise Clusters for Catalysis. *Nanoscale Adv.* **2020**, *2*, 55–69.

(10) Ma, Y.; Yu, G.; Wang, T.; Zhang, C.; Huang, X.; Chen, W. Highly Efficient Catalytic Activity for the Hydrogen Evolution Reaction on Pristine and Monovacancy Defected WP Systems: A First-Principles Investigation. *Phys. Chem. Chem. Phys.* **2018**, *20*, 13757–13764.

(11) Vayssilov, G. N.; van Santen, R. A. Catalytic Activity of Titanium Silicalites—a DFT Study. *Journal of Catalysis* **1998**, *175*, 170–174.

(12) Zhou, M.; Zhang, A.; Dai, Z.; Zhang, C.; Feng, Y. P. Greatly Enhanced Adsorption and Catalytic Activity of Au and Pt Clusters on Defective Graphene. *J. Chem. Phys.* **2010**, *132*, 194704.
